# The effects of Hh morphogen source movement on signaling dynamics

**DOI:** 10.1242/dev.199842

**Published:** 2022-12-07

**Authors:** David G. Míguez, Antonella Iannini, Diana García-Morales, Fernando Casares

**Affiliations:** ^1^ Departmento de Física de la Materia Condensada, Instituto de Física de la Materia Condensada (IFIMAC), Facultad de Ciencias, and Centro de Biología Molecular Severo Ochoa (CBMSO, CSIC-UAM), Universidad Autónoma de Madrid, 28049 Madrid, Spain; ^2^ Gene Expression and Morphogenesis Department, CABD (Andalusian Centre for Developmental Biology), CSIC/Universidad Pablo de Olavide/Junta de Andalucia, Campus UPO, 41013 Seville, Spain

**Keywords:** Hh signaling, *Drosophila*, Morphogens, Computational modeling, Dynamical systems

## Abstract

Morphogens of the Hh family trigger gene expression changes in receiving cells in a concentration-dependent manner to regulate their identity, proliferation, death or metabolism, depending on the tissue or organ. This variety of responses relies on a conserved signaling pathway. Its logic includes a negative-feedback loop involving the Hh receptor Ptc. Here, using experiments and computational models we study and compare the different spatial signaling profiles downstream of Hh in several developing *Drosophila* organs*.* We show that the spatial distributions of Ptc and the activator transcription factor CiA in wing, antenna and ocellus show similar features, but are markedly different from that in the compound eye. We propose that these two profile types represent two time points along the signaling dynamics, and that the interplay between the spatial displacement of the Hh source in the compound eye and the negative-feedback loop maintains the receiving cells effectively in an earlier stage of signaling. These results show how the interaction between spatial and temporal dynamics of signaling and differentiation processes may contribute to the informational versatility of the conserved Hh signaling pathway.

## Teaser

**Summary:** A combination of computational modeling and experiments examining the spatial distribution of Ptc and CiA reveals distinct signaling profiles downstream of Hh in different organs during *Drosophila* development.

## INTRODUCTION

**Fig. 1.**
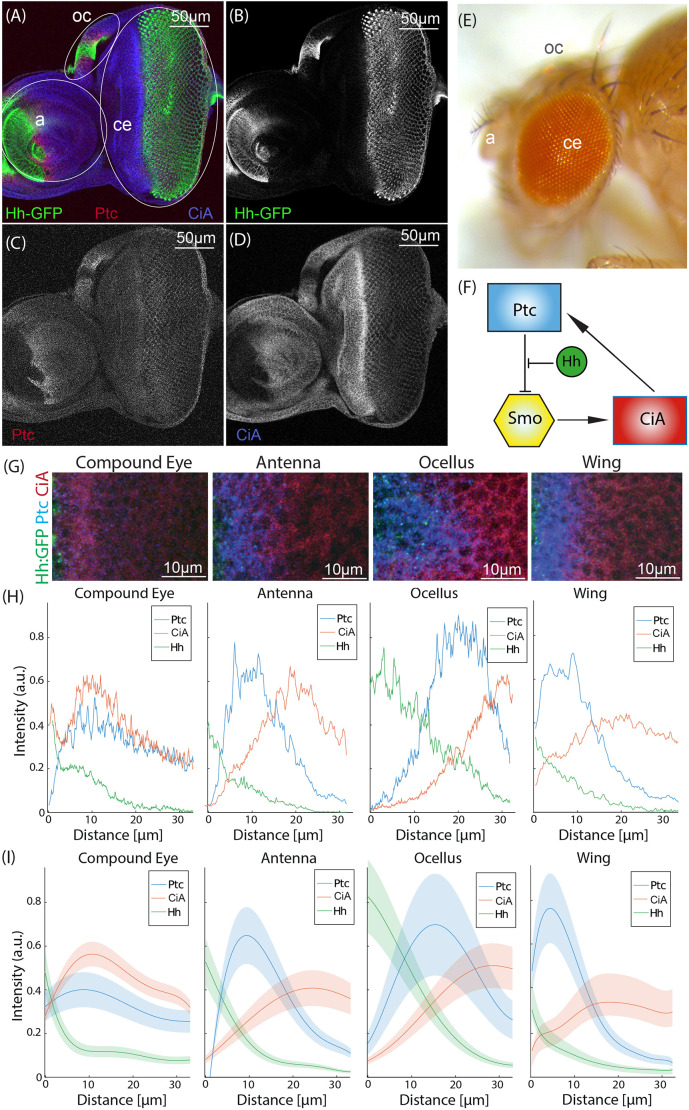
**Hh signaling profiles in different organs.** (A-D) Confocal image of a Hh:GFP eye-antennal disc stained for GFP, Ptc and CiA. Merged (A) and individual signals (B-D) are shown. (E) Adult *Drosophila* head showing the adult derivatives of the eye-antennal disc: CE, antenna and ocelli. a, antenna; ce, compound eye; oc, ocelli. (F) Basic architecture of the Hh signaling pathway. In the absence of Hh signal, Ptc inhibits the production of CiA by Smo. By binding to Ptc, Hh relieves this inhibition. The production of CiA results in the transcriptional activation of the pathway's targets. One of these targets is Ptc itself. (G) Representative snapshots of CE, antenna, ocellus and wing discs stained for Ptc (blue), CiA (red) and Hh:GFP (green), spanning the region abutting the Hh-expressing domain from late-L3 larvae. (H) Representative values of the spatial distribution of the levels of Ptc (blue), CiA (red) and Hh (green) for the four snapshots above. The Hh:GFP source is located at distance=0. (I) Average values of the different staining profiles for at least five independent replicates for each tissue (see Table S1 for measurements used). Shaded areas represent 50% confidence interval. Images shown in G are representative of at least five samples.

The development of almost all organs of any animal relies on Hh signaling for the control of their growth, differentiation and/or patterning. These roles extend beyond early development into the adult, where the Hh pathway is required for tissue homeostasis and regeneration. In addition, mutations affecting components of this pathway contribute to a number of congenital diseases and cancer types (Ingham et al., 2011). Although the biochemical and cellular details of Hh ligand production, distribution and receptor binding, and signal transduction events downstream of Hh are extremely intricate (and to date not fully elucidated), the Hh signaling pathway seems to be governed by a universal underlaying regulatory logic (Kong et al., 2019; Li et al., 2018; Fig. 1). The major output downstream of Hh is the regulation of gene transcription in receiving cells (Briscoe and Therond, 2013; Ingham et al., 2011; Lee et al., 2016; Petrova and Joyner, 2014). The transcription factors in charge of this transcriptional regulation belong to the Gli family, which in *Drosophila* are represented by the single *ci* (*cubitus interruptus*) gene. In the absence of signal, Ci/Gli undergoes proteolytic cleavage into a transcriptional repressor (in *Drosophila* it is named ‘CiR’). Hh, binding to its receptor Ptc/PTCH, relieves Ptc repression on Smo. Free from this repression, Smo blocks the proteolysis of Ci/Gli to render a full-length Ci/Gli that acts as a transcriptional activator (‘CiA’ in *Drosophila*) (Aza-Blanc et al., 1997). Interestingly, Ptc is among the transcriptional targets of the pathway: cells receiving Hh upregulate *ptc* expression (Fig. 1F; Capdevila et al., 1994; Tabata and Kornberg, 1994). As a result of this feedback, Hh binding by increasing levels of Ptc dynamically alters the quantitative profile of Hh across the field of Hh-receiving cells and modifies the signaling levels in those cells (Briscoe et al., 2001; Chen and Struhl, 1996; Garcia-Morales et al., 2019; Li et al., 2018; Miguez et al., 2020). Hh proteins are produced in specific domains within a developing organ and lay adjacent to Hh-responsive cells expressing basal levels of *ptc* and *ci*. The Hh-producing cells are often insensitive to it by maintaining repression of *ptc* and *ci* (Sanicola et al., 1995; Schwartz et al., 1995). Responsive cells alter their gene expression according to the intracellular signaling levels elicited by the concentration of Hh they receive. As a result, different gene expression patterns are established at different distances from the Hh source, resulting in patterned cell behavior. These are the characteristics defining a ‘morphogen’ (as defined by L. Wolpert; Wolpert, 1969). This morphogen-type action has been characterized in a number of systems, including the *Drosophila* wing primordium –usually called wing ‘disc’ (Mullor et al., 1997; Strigini and Cohen, 1997).

Another organ in *Drosophila* that requires Hh for its development is its compound eye (CE; Fig. 1A-D). In this organ, once the first photoreceptor R-cells are induced to differentiate by the BMP2/3 Dpp produced by the cells surrounding the eye primordium (Bras-Pereira et al., 2006; Treisman and Heberlein, 1998), they start expressing Hh (Lee et al., 1992). Hh, dispersing from these R-cells, activates the expression of the proneural gene *atonal* (*ato*) in cells immediately anterior to them. *ato* then triggers a series of regulatory steps that lead ultimately to the differentiation of new R-cells (Dominguez, 1999; Jarman et al., 1995). As part of their differentiation process, these new R-cells become sources of Hh as well. This positive-feedback loop results in a characteristic wave of differentiation (Miguez et al., 2007) that sweeps across the primordium until the pool of progenitor cells is exhausted (reviewed by Miguez et al., 2020). Therefore, in its eye function, rather than generating spatially patterned cell diversity, Hh induces only one major cell fate transition (a proneural fate) reiteratively. This feature, together with the fact that the source of Hh is moving as differentiation proceeds, contrasts with the situation in the wing, where the source of Hh is spatially static and acts as a prototypical morphogen.

This inherent versatility of the Hh pathway, which allows it to drive the differentiation of organs as different as wings, eyes or antennae, is still not fully understood. Here, we combine modeling and experiments to investigate this question by comparing the dynamics and signaling profiles downstream of Hh in different organs during *Drosophila* development. Our results suggest that the interplay between the dynamics of the negative feedback between Hh and Ptc and the dynamics of the Hh source provides the versatility to produce the different signaling signatures required to drive patterning in different organs.

## RESULTS AND DISCUSSION

We started by characterizing quantitatively Hh signaling in different developing fly organs. To do this, we used confocal microscopy to measure simultaneously the levels of Hh (signal) across fixed eye-antennal and wing discs of late third-stage (L3) larvae, together with the expression profiles of two signaling readouts: Ptc (receptor as well as repressor and transcriptional target of the pathway) and the activator form of Ci, CiA (transcriptional activator) (Fig. 1A-D). The eye-antennal disc gives rise to the CE as well as the antenna and the ocelli (small single-lens eyes located on the forefront of the head) (Fig. 1E; Haynie and Bryant, 1986). Each of these organs expresses Hh (Fig. 1A-D) and requires Hh signaling for its development (Heberlein et al., 1993; Ma et al., 1993; Mohler, 1988; Royet and Finkelstein, 1997). To obtain the Hh profile, the larvae carried a bacterial artificial chromosome (BAC) containing a functional GFP-tagged Hh gene (Chen et al., 2017), so that the GFP signal is a faithful reporter of Hh protein distribution. Ptc and CiA were detected using specific antibodies (see Materials and Methods) (Fig. 1G). In all four organs, the Hh:GFP signal exhibited a declining profile from the source, as expected (Fig. 1H,I). However, we noted two things. First, the signaling profiles of Ptc and CiA in the wing, antenna and ocellus were qualitatively similar, with a first peak of Ptc followed by a peak in CiA further from the Hh source. By contrast, the signaling signature of Hh in the CE was qualitatively different from the others, with a peak expression of Ptc that was coincident in space with a maximum in CiA. A second main difference was that the signal intensity of Ptc at this maximum was significantly lower in the CE than in the other tissues.

**Fig. 2.**
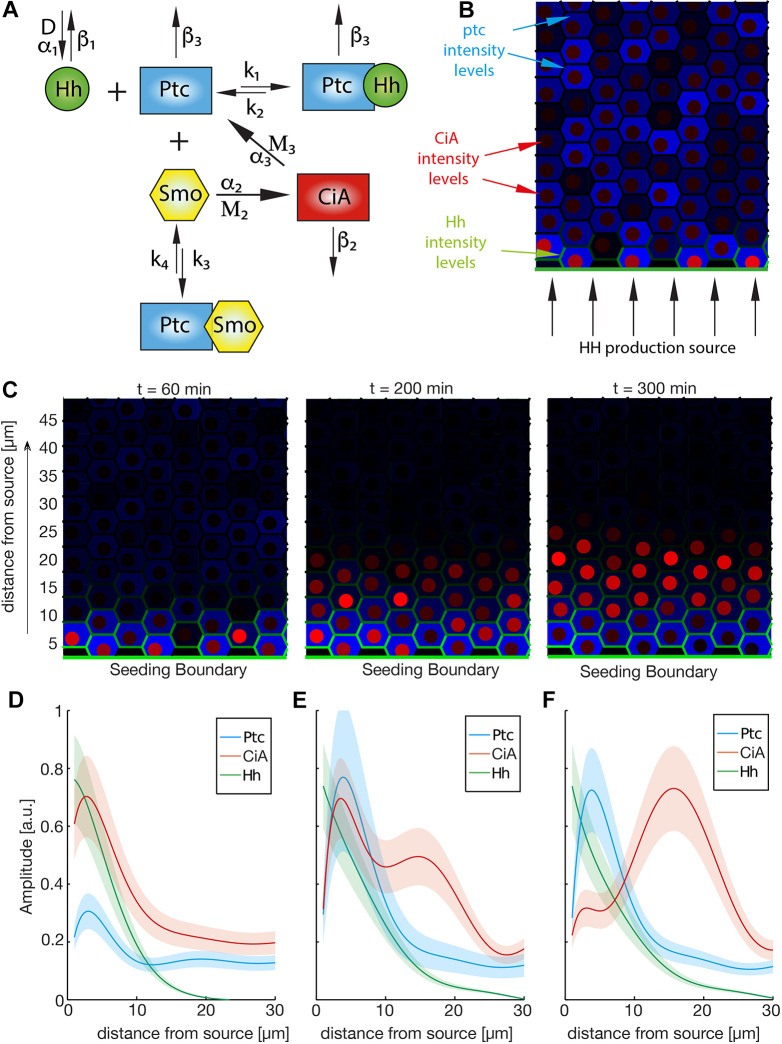
**Computational model of the Hh signaling pathway.** (A) Scheme of the interactions of the numerical model, with the parameters used. (B) Spatial configuration of the model as a 2D array of 10×10 cells. The Hh source is located at the lower boundary. Protein levels in each cell are indicated by color intensities. For visualization purposes, we represent Ptc levels in blue in the cytoplasm, Hh levels in green at the membrane, and CiA levels in red in the nucleus (see supplementary data file 1 for code of 2D model). (C) Output of a single simulation at different times, in terms of the total levels of Ptc, CiA and Hh at different time points. (D-F) Spatial profiles of Ptc, CiA and Hh computed based on distance of each cell to the Hh source. Lines represent average values of fitted curves for five independent numerical simulations. Shaded areas represent 50% confidence interval.

The observation of non-coincident peaks for CiA and Ptc in wing, antenna and ocellus might not have been surprising in a spatially extended system with Ptc exerting negative feedback, but why is it not observed in the CE? One possibility to explain these organ-specific differences in signaling profiles is that the Hh core signaling pathway is subject to organ-specific modifications. An alternative explanation is that the two signaling signatures of Hh can emerge without the need of organ-specific modifications. To explore this second possibility, we built a computational model of the Hh core signaling pathway, as outlined in Fig. 1F, and modeled as indicated in Fig. 2A.

The model was first formulated as a system of ordinary differential equations (ODEs) that capture the core pathway: the binding of a diffusible Hh to its receptor Ptc relieves the repression that Ptc exerts on Smo. This latter, when freed, activates Ci to produce the transcriptional activator, CiA, which, in turn, feeds back onto the pathway by activating Ptc production (Fig. 2A; see Materials and Methods for details of the modeling equations).

To study the generation of the signaling profiles in the tissue, we extended our model as a spatial and temporal hybrid by embedding the ODEs in a spatial array of 10×10 hexagons representing the bidimensional epithelial cell layer of the fly organs. An example of this computation is shown in Fig. 2B. The temporal scale was calibrated using measurements taken by Nahmad and Stathopoulos (2009), in which the authors estimated the time required for maximal activation of Ptc by Hh in the wing disc (360 min at 18°C). Because developmental time at 18°C approximately halves at 25°C (Powsner, 1935), the temperature at which our experiments were carried out, the model parameters were calibrated to produce maximal Ptc activation at around 180 min after Hh stimulation (see Materials and Methods).

When we ran the simulations and followed the profiles over time (Fig. 2C, Movie 1), we observed that, at earlier times, the Hh gradient that is formed drives CiA and Ptc with peaks in similar positions, close to the source of the gradient. However, as time proceeds, CiA maximum is displaced as Ptc expression increases close to the source (Fig. 2D-F). Therefore, the model reproduces the profiles for the CE and the other organs as an early and a later time point, respectively, along its temporal dynamics. The peak displacement can be explained by our model as a consequence of the delay between CiA activation and the feedback mediated by Ptc: initially, Hh reception leads to CiA production followed by an increase in Ptc transcription. But, as time passes and Ptc expression increases, its negative action on the pathway (inhibiting Smo) results in the reduction of CiA levels close to the source. As the Ptc profile sharpens close to the source, the non-bound Hh ligand keeps dispersing farther and activates CiA, which now shows a peak beyond that of Ptc. At this point, the system reaches a quasi-stationary state that strongly resembles the experimental condition measured in the wing, antenna and ocellus (Fig. 1H,I). This profile arises for a wide range of values of the model parameters, as shown in Fig. S4.

**Fig. 3.**
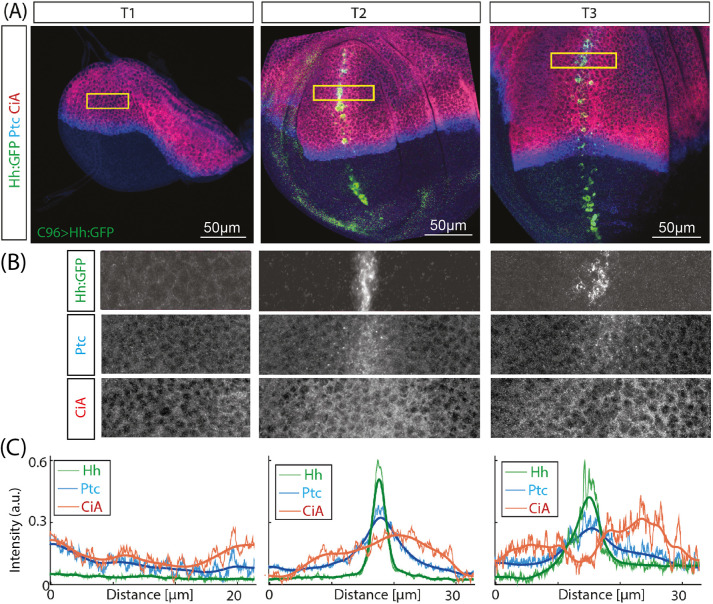
**Progressive separation of Ptc and CiA signal maxima in wing discs with ectopic Hh expression.** (A) Expression of *UAS-Hh-GFP* driven by *c96-GAL4* along the dorsoventral wing disc boundary initiates in mid-L3 discs (T2). At earlier time points, there is no Hh-GFP expression (T1). Representative wing discs are shown. Expression profiles for Ptc and CiA were obtained from similar regions of the anterior compartment of the wing disc pouch (boxed) from progressively older discs (T1-3). (B,C) Hh-GFP, Ptc and CiA expression (B) and quantitative profiles (C) from the boxed region in A in early (T1), mid (T2) and late (T3) wing discs centered around the *c96-GAL4* expression domain. The actual profiles and the denoised curves (thicker lines) are shown. After initiation of induction, there is an increase in CiA and Ptc expression with coincident maxima straddling the Hh-GFP domain (T2). In the T3 disc, the CiA maxima are displaced laterally relative to the Ptc peak (T3). Images shown in B are representative of at least five samples.

To test the computational prediction that, upon Hh reception, Ptc and CiA peaks first overlap and then the CiA peak is displaced, we expressed a GFP-tagged form of Hh (Hh-GFP; Callejo et al., 2008) in the anterior region of the wing disc (where Hh is not produced, but received) and followed the response of the Ptc and CiA signals (Fig. 3). As driver, we used the *c96-GAL4* line, expression of which initiates in mid-third larval stage (mid-L3; see Materials and Methods) in a stripe along the dorso/ventral boundary of the wing pouch (Fig. 3A). In mid-L3 discs, around the time of Hh-GFP expression initiation, the Ptc and CiA signals increased, straddling the Hh-GFP cells. However, in late-L3 discs, although the Ptc peak remained centered on the Hh-GFP domain, the CiA maxima were displaced (Fig. 3B,C). Therefore, this ectopic-expression assay reproduces the temporal dynamics of the Ptc and CiA profiles predicted by the model.

**Fig. 4.**
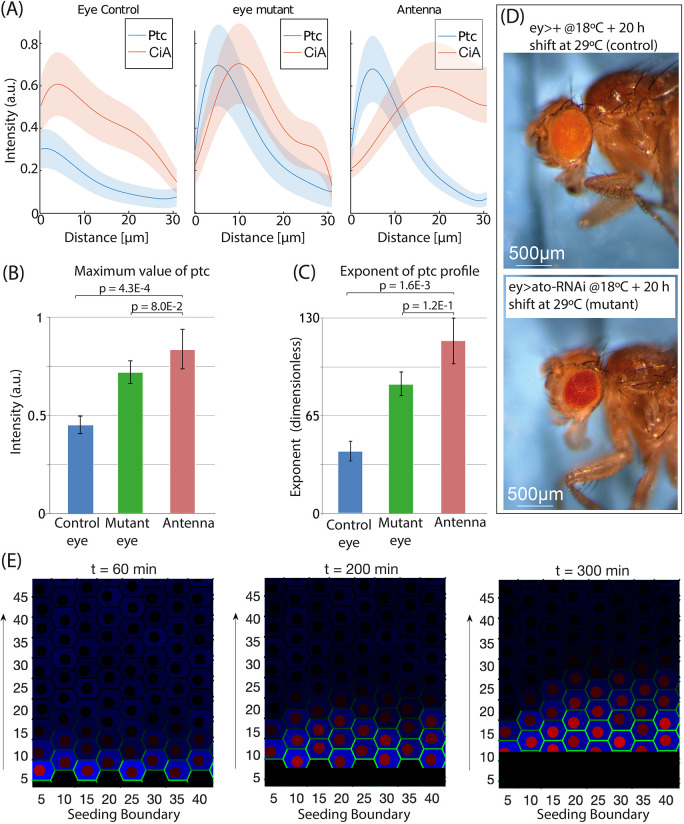
**Ptc and CiA profiles in eye discs with a slow differentiation wave.** (A) Average spatial distribution of the different profiles of Ptc and CiA (at least four independent discs) for control and mutant (*ato-RNAi* expression pulse; see main text and D) CE and antenna. Shaded areas represent 50% confidence interval. (B) Quantification of maximum Ptc values in these three conditions. (C) Quantification of exponent of the exponential fitting of the decaying region in the Ptc profile, for the three conditions tested. Statistical analysis (two-sample, unequal, one-tailed Student's *t*-test) shows that the differences between the mutant and the antenna are not significant, but are highly significant when the control and mutant CEs are compared. Error bars indicate s.e.m. (D) Adult control (top) and *ato-*mutant (bottom) flies. The transient stalling of the differentiation process results in smaller eyes. See main text for detailed explanations of the experiment. (E) Computational experiment in which the Hh-producing front moves. *y*-axis shows distance from the source (μm). As in Fig. 2, Hh is green, Ptc is blue and CiA is red. Snapshots at 60, 200 and 300 min are shown. The maxima of CiA and Ptc expression are coincident even at 300 min. Quantification is shown in Fig. S2.

All these results pointed us to investigate further why the signaling profile of the CE appears to be at an earlier stage of this dynamics. As described above, the Hh source is spatially static in wing, antenna and ocellus, whereas it moves in the CE. If in the CE the receiving cells, as a result of the differentiation wave, became an Hh source faster than the time required for the activation of the negative-feedback loop that separates the Ptc and CiA peaks, the Hh signaling profile would not progress beyond an ‘early stage’ profile. This would imply that different signaling profiles in different organs could simply emerge from the signaling dynamics without the need for organ-specific modifications of the pathway to explain them. To test this hypothesis, we carried out an experiment in which we delayed the movement of the differentiation wave (Fig. 4). To do this, we expressed an RNAi targeting specifically *ato* in the developing CE for a period of 20 h, after which we dissected the eye-antennal discs and profiled the expression of CiA and Ptc (see Materials and Methods). By attenuating *ato*, the process of R-cell recruitment should be delayed or halted, as *ato* is required for the specification of the founder R-cells, the R8 type (Jarman et al., 1995). Indeed, the adults from this experiment had smaller eyes (Fig. 4D). We could estimate the reduction of the morphogenetic furrow (MF) velocity as the percentage of ommatidial rows assembled in mutant CE discs (*ey>atoRNAi*) relative to those in controls at the end of L3, assuming a constant speed in both groups. The number of ommatidia in late L3 CE discs was 17±2 and 6±3 (mean±s.d.) in controls and mutants, respectively (*n*=10 for each group). Therefore, the MF speed was reduced, on average, to about one-third of the control. This means that, instead of 60-100 min at 25°C (Basler and Hafen, 1989), the recruitment of one cell row by the signaling wavefront should take 3 h or longer in our mutant discs. As mentioned above, the full Hh signaling, including the feedback-induced Ptc upregulation, should take about 3 h. Therefore, the retardation in wavefront movement in mutant CE discs should allow approximately the time needed for the Hh signaling to complete its feedback, visible as the Ptc/CiA peak separation. Indeed, when we compared the CiA and Ptc profiles in control and *ato-*RNAi (‘mutant’) discs, the profiles of mutant CEs were intermediate between those of control CEs and of antennae (as the antennae were not affected by the eye-specific manipulation, they showed profiles indistinguishable between control and mutants; Fig. 4A). In the *ato-*RNAi CE, the Ptc profile showed increased maximal amplitude and a steeper slope, both differences statistically significant, plus a slight displacement of the CiA peak compared with control CEs (Fig. 4B,C). This profile is equivalent to the computational profile at intermediate times (see Fig. S2) and therefore supports our hypothesis that the two types of signaling profiles arise from the same signaling network and depend on whether the Hh source is spatially static or not.

To test this hypothesis further, we used again our computational model. In this experiment, we repeated the same conditions and parameter values, with the only difference being that now we set the Hh source to move across the cell field at a speed of one cell per hour. Fig. 4E shows three snapshots of this scenario taken at three different time points (a time-lapse movie is presented as Movie 2, to be compared with the scenario of a static Hh source presented in Movie 1). The simulation shows that, when the source of Hh is moving across the tissue, the separation between the CiA and Ptc peaks does not occur, which recapitulates the profiles obtained experimentally in the CE (Fig. 1G-I). This contrasts with the result of a static source, where the same parameters and conditions result in a clear separation of the maxima at 300 min (Fig. 2C-F), similar to the profiles of antenna, ocelli and wing primordia (Fig. 1G-I). Using the computational model, we investigated how sensitive the Ptc/CiA peak separation signature was relative to the speed of the Hh source (Fig. S3). Already with velocities as slow as 0.1 cell diameters per hour (10% of the normal speed) the peak separation was not observed (Fig. S3). Therefore, the model result suggests that Hh signaling dynamics is very sensitive to the movement of the Hh source, which is in agreement with our experimental results, in which peak separation in the CE begins to be observed when source speed is experimentally reduced to about a third of normal. Finally, the model was used to explore how the expression pattern depends on the relative time scales of the feedback loop and morphogen diffusion. Results summarized in Fig. S5A show that the distance between CiA and Ptc peaks increases as the dynamics of the feedback decreases, because it allows the Hh gradient to reach cells further from the source. We also used the model to test whether differences in Ptc production could explain the different profiles in CE versus the other tissues. Simulations in which the production rate of Ptc was reduced (Fig. S5B) showed that the peak separation still occurs, but at longer times (which makes sense, given that the dynamics is slower). These results stress the idea that the temporal dynamics of the signaling pathway and the separation of the peaks of the crucial molecules, Ptc and CiA, depend on the relationship between two temporal scales: those of Hh diffusion and the negative feedback.

In conclusion, we show that the organ-specific Hh signaling profiles are just different states along the temporal dynamics of the pathway. In the CE, Hh-receiving cells start to differentiate into Hh-producing cells faster than the time required for downstream interactions to reach a steady state, which is attained in other tissues in which the Hh source is spatially static. These results do not exclude potential organ-specific modifications of the Hh signaling pathway and its dynamics. For example, it has been described in the CE that the nuclear factor Dachshund (Dac), the expression of which depends on Hh signaling, is required for the stabilization of CiA (Bras-Pereira et al., 2016), which might additionally contribute to fine-tuning of the pathway dynamics in this organ.

The two signaling profiles that we have described, one characterizing the CE and the other shared by all the other organs analyzed, likely differ in the information they carry. The wing/antenna/ocellus profile defines two clearly different signaling peaks, which may be further enriched by the temporal dynamics of the signaling before reaching a steady state. In the CE, however, the profiles suggest that once cells start receiving Hh signaling, they increase their signaling until they are taken over by the differentiation wave (and initiate their differentiation that transform them from Hh-receiving to Hh-producing cells). Therefore, the difference in signaling profiles we have noted may thus reflect different functional ‘modes’. In the ‘spatial mode’, Hh generates an information-rich, spatially static gradient that allows the definition of several distinct positional values, such as those Hh instructs in the wing, and therefore works as a morphogen. In the CE, Hh works in a ‘mobile mode’: the moving gradient is less rich in spatial information as it is used to trigger a single cell fate repeatedly across a field of cells. In this mode, Hh works as a fate ‘inducer’, rather than as a prototypical morphogen. In this mobile mode, the speed at which the Hh source travels depends on the kinetics of the biochemical steps triggered by the reception of the signal initiating photoreceptor cell differentiation and their production of Hh, resulting in the coupling between gradient movement and cell differentiation.

## MATERIALS AND METHODS

### *Drosophila* strains and genetic manipulations

The Hh:GFP strain has been described by Chen et al. (2017). They carry a BAC harboring a functional tagged *hh* locus. This is a rescuing line, i.e. it is able to rescue a null *hh* genetic condition, and therefore Hh:GFP can be confidently considered a good reporter of the normal Hh protein distribution. To drive Hh expression in the wing disc, we used the *c96-GAL4* driver (Gustafson and Boulianne, 1996) and the *UAS-Hh-GFP* responder line (Callejo et al., 2008). Specifically, we used the *c96-GAL4* driver in combination with a ubiquitously expressed temperature-sensitive GAL80 form (GAL80ts). The larval progeny of the *c96-GAL4, GAL80ts* × *UAS-Hh-GFP* cross was grown at constant 16°C to weaken and delay the onset of Hh-GFP expression, as preliminary experiments showed that at higher culture temperatures Hh was induced in very early discs making it more difficult to dissect discs before the onset of *c96-GAL4*-induced Hh-GFP expression.

To attenuate *atonal* (*ato*), larvae from the cross *ey-GAL4* (FlyBase: https://flybase.org/reports/FBtp0002646) females to *UAS-atonal RNAi* (Vienna Drosophila Resource Center #49675; Dietzl et al., 2007) males were raised at 18°C for most of their development. The GAL4/UAS system is temperature sensitive, with increasing expression at higher temperatures. At 18°C, the eyes of the resulting adults were indistinguishable from controls (not shown). To induce the expression of the *ato-*RNAi construct, larvae were transferred at 29°C and dissected 20 h afterwards. We also performed the analysis at later times (∼30 h at 29°C), but at this point the CiA and Ptc signals were very low anterior to the stalled retina, likely because of increased cell death (not shown), so the profiles were not used. Adult flies were imaged using a Leica 490 digital camera on a Leica DFC 320 binocular scope.

### Confocal microscopy and image analysis

Immunolocalization and confocal microscopy was performed as described by Garcia-Morales et al. (2019). Antibodies used were: mouse anti-Ptc (Apa1; 1/100), rat anti-CiA (2A1; 1/5) and rat anti-Elav (9F8A9; 1/2000) (obtained from the Developmental Studies Hybridoma Bank, created by the NICHD of the NIH and maintained at The University of Iowa, Department of Biology, Iowa City, USA) and rabbit anti-GFP (A11122, Molecular Probes; 1/1000). The anti-Elav antibody is a pan-neural marker that recognizes ommatidial photoreceptors (O'Neill et al., 1994). Alexa Fluor-conjugated secondary antibodies, diluted at 1/400, were from Molecular Probes (Alexa 488 anti-rabbit, A11034; Alexa 568 anti-mouse, A11031; Alexa 647 anti-rat, A21247). All discs were from late-L3 larval stages, except for those in Fig. 3, which were sampled throughout the L3 stage. Confocal images were collected as *z*-stacks and processed using Fiji (Schindelin et al., 2012). Strips of tissue were oriented such that they were orthogonal to the border of the Hh source, which in the CE corresponds to the MF. These strips were further trimmed to exclude source cells. Then, two to four optical *z*-sections, selected as containing most of the signal per oriented and trimmed stack were projected using the ‘maximal projection’ tool and the signal for each of the three channels (each representing one of the three proteins, Hh:GFP, Ptc and CiA) was measured and extracted as a spatial profile. To register the profiles, *x*=0 was defined as the point where levels of Hh:GFP started to decrease, marking the beginning of the domain of Hh-receiving cells.

Ommatidial counts were carried out on the confocal images of eye discs from the same samples, which were co-stained with Elav, as the number of Elav-positive cell clusters counted from the posterior margin (around the position of the optic nerve).

### Computational model

The system of equations representing the interactions modeled are:
(1)

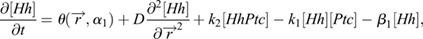

(2)

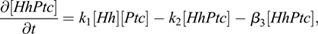

(3)

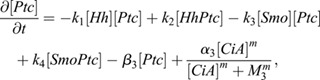

(4)

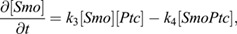

(5)

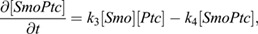

(6)

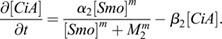


Eqn 1 represents the dynamics of Hh, with *D* being its diffusion coefficient and ø(r, α_1_) a function that defines the location of Hh production and its level at the source (α_1_). In this way, the Hh source can be defined as steady or moving depending on the simulation requirements. The next terms of the equation account for the binding and unbinding of free Hh and free Ptc (rate constants *k*_1_ and *k*_2_, respectively) forming the complex HhPtc, with β_1_ representing the constant degradation of the free Hh molecule. The level of Hh for each cell is calculated as the average value of the diffusing Hh across the area of each individual cell.

Eqn 2 models the formation dynamics of the complex between Hh and Ptc, with degradation constant β_3_. Eqn 4 corresponds to the dynamics of free Ptc, which can also bind to and unbind from Smo (rate constants *k*_3_ and *k*_4_), and degradation constant β_3_ (similar to HhPtc). The last term represents the activation of free Ptc production by CiA as an activating Hill function with Hill coefficient *m* and concentration threshold *M*_3_. Eqns 5 and 6 correspond to the dynamics of free (‘Smo’) and bound (‘SmoPtc’) configurations for Smo. For Smo, we assume a non-zero, constant concentration across the tissue, only undergoing transitions between the unbound and Ptc-bound forms (see below). Eqn 6 illustrates the production of CiA by Smo, also as a simple Hill function with rate constant α_2_ and Hill coefficient *m* and concentration threshold *M*_2_. The Hill function is used here to simplify the dynamic balance between the activator CiA and repressor form CiR mediated by Smo, so we can eliminate directly the variable CiR from the equations. This simplification is based on the idea that dynamics of the balance between the two forms is fast (binding-unbinding), compared with the other transcription process, so the two time scales can be separated and the levels of CiA can be considered in equilibrium, reducing the number of equations and the number of parameters. Finally, CiA is assumed to degrade with rate constant β_2_.

Numerical simulations of the model equations were performed using a computational script written in MATLAB (MathWorks) and developed in-house (available as supplementary data file 1). In the model, space and time are discretized using the Euler algorithm. Concentrations are considered as dimensionless, but space and time are dimensional (µm and min, respectively). The model consists of a hybrid approach that combines ODEs for the processes inside cells and partial differential equations for processes across cells, such as morphogen transport. Cells are simulated as 2D hexagonal regions in a Voronoi diagram.

Values for the initial concentrations of the variables and for each of the model parameters and for each cell in the tissue are obtained from a gamma distribution with standard deviation set to 10% of the mean value, to represent cell variability (mean value for each parameter is listed in Table 1). Values for Hh concentration are stored in a 2D matrix. For each time step, the algorithm proceeds as follows: (1) Hh concentration is modulated by its production at the source and diffusion in the 2D space (values of the 2D matrix are updated); (2) the amount of Hh that each cell receives is computed; (3) dynamics for each cell is computed based on the Hh that it is sensing; (4) the concentration of Hh changes locally as a result of interactions with the receptor (binding, unbinding, degradation); (5) the values in the 2D matrix of Hh concentration are updated to account for these local interactions (so, levels of the diffusing molecule are determined by the events in each cell); (6) back to step (1).

**Table 1.**
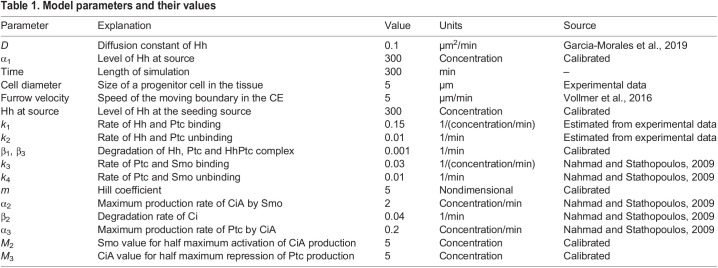
Model parameters and their values

Finally, to obtain the profiles of expression levels, the values for the total amount of each variable were computed and averaged based in the distance of each cell to the source (located at *x*=0). This sequence of events was performed for each time point during the simulation.

The source of Hh was set in our system as the lower boundary (labeled as ‘Seeding Boundary’ in the plots). From here, Hh enters the spatial system and its transport is simplified as standard 2D diffusion. In numerical simulations that involve a moving morphogenetic furrow, this is introduced by translocation of the lower boundary of the system (which is continuously secreting the Hh protein). This way, the boundary is set to move at constant speed upwards traveling across the system. Cells that have been ‘run over’ by the moving furrow boundary are set to differentiate and stop consuming Hh (colored in black in the simulation, for visualization purposes).

Preliminary versions of the model that included explicitly the dynamics of production and degradation of Smo showed an initial transient that quickly reached a constant concentration (Smo production and degradation are not part of the feedback). Therefore, for the sale of simplicity, the model was simplified assuming constant levels of total Smo.

### Spatial and temporal calibration

To reduce the number of free parameters and equations, and to obtain a simpler model that could be more easily explored, we opted to condense several features of the Hh pathway in a small set of interactions. Therefore, some parameter values cannot be directly informed from the literature, because they encompass multiple interactions. The correct ranges of some parameter values have to be estimated by comparison with the dynamics and steady state values of the real system.

Table 1 shows the values of the model parameters used in our simulations. For the spatial calibration, we estimated the size of a cell at around 5 µm in diameter (estimated from the experimental snapshots).

For the Hh diffusion, we use a value *D* of 0.1 µm^2^/min, which is similar to the values obtained from experimental measurements in the literature (Garcia-Morales et al., 2019), and which produces an exponential profile of characteristic length of around 12 µm in steady state. We have measured previously that the characteristic length of the Hh exponential profile is around 13 µm and that is conserved for antenna, ocellus, CE and wing disc (Miguez et al., 2020). This value of Hh diffusion, combined with the value of Hh production at the source and degradation rates (β_1_ for Hh) also result in a steady state configuration reached at around 60 min in the absence of other downstream interactions, which is consistent with our previous data (Garcia-Morales et al., 2019).

The calibration of the time variable in the model is based on experimental data from Nahmad and Stathopoulos (2009). Here, authors estimate that Ptc levels reach their maximum after 360 min at 18°C. Dynamics at standard culture conditions (25°C) is twice as fast as the dynamics at 18°C, so we estimate that the increase of Ptc in standard conditions occurs at 180 min. Based on these data, we set our parameter values to obtain a peak in Ptc levels at around 180-200 min after Hh stimulation. This time reference was used to estimate the equivalence between time step in the simulation and minutes in the experimental system.

In terms of the shape and relation between Hh, Ptc and CiA profiles, the model output is robust to large variations in the value of the Hill coefficients; therefore, an intermediate value for the slope of the Hill function was chosen for the final simulations (*m*=5). The value of *M*_2_ and *M*_3_ (for half maximum activation and repression of CiA and Ptc, respectively), were estimated by performing numerical simulations and monitoring the maximum and minimum levels of the substrate molecule in each case. Then, the value of *M*_2_ and *M*_3_ was set as the middle point between these maximum and minimum values. This way, we calibrated the transition between no activation to activation (or no repression to repression) to occur inside the range of values of the substrate molecule. For *M*_2_, the range where we observed a clear peak separation was between 3.3 and 7.5 (around 50% above and below the value used of 5). For *M*_3_, the range was 1.6-50 (four times smaller and ten times larger than the value of 5 used).

Finally, affinity and dissociation of Hh and Ptc were estimated from previous experimental data, whereby it was calculated that, in steady-state conditions, around 90% of the Ptc is bound to Hh, and that this percentage is independent of the distance to the Hh source (Miguez et al., 2020).

To illustrate the robustness of the observations, we performed numerical simulations with variations of 50% above and below some of the key parameters (summarized in Fig. S4).

The parameters of the model and their values can be found in Table 1.

### Statistical analysis

Average profiles for the expression levels of Hh, Ptc and CiA for experiments were obtained by polynomial fitting of the experimental profiles of at least five profiles for each condition. Polynomial fitting of each profile allowed us to perform statistics between experimental profiles of different size and/or spatial resolution. Comparison of the average using polynomial fitting and the direct point-to-point average is shown as Fig. S1, showing a similar trend. Average values for the profiles were obtained by 7-order polynomial fitting, using the package LsqFit from Julia. Polynomial fitting was performed using the ‘curve_fit’ function. Residuals for each polynomial fitting are included in Table S1.

Next, we calculated the mean and the standard error for each parameter of the polynomial fitting. Finally, we constructed a new polynomium using these average parameters, and represented the resulting curve as the average profile for each signal and each condition.

Experimental spatial profiles were obtained by selecting a rectangular region in the sample. The width of the rectangular regions was three to four cell diameters, and the length was varied depending on the tissue being imaged. The spatial profile was obtained as the signal intensity average across the width.

For the simulations, the individual profiles were obtained in a similar fashion as in the experiments. In brief, the values of the expression levels for Hh, Ptc and CiA were averaged over cells that were at a similar distance from the Hh source, transforming in this way information from a 2D array of cells to a 1D profile.

## Supplementary Material

10.1242/develop.199842_sup1 Supplementary information
