# Supplementary material for: The effects of Hh morphogen source movement on signaling dynamics
Source: Development. 2022 Dec 7;149(23):dev199842. doi: 10.1242/dev.199842 (PMC10114110; doi:10.1242/dev.199842)
Supplement: Supplementary information [file develop-149-199842-s1.pdf]

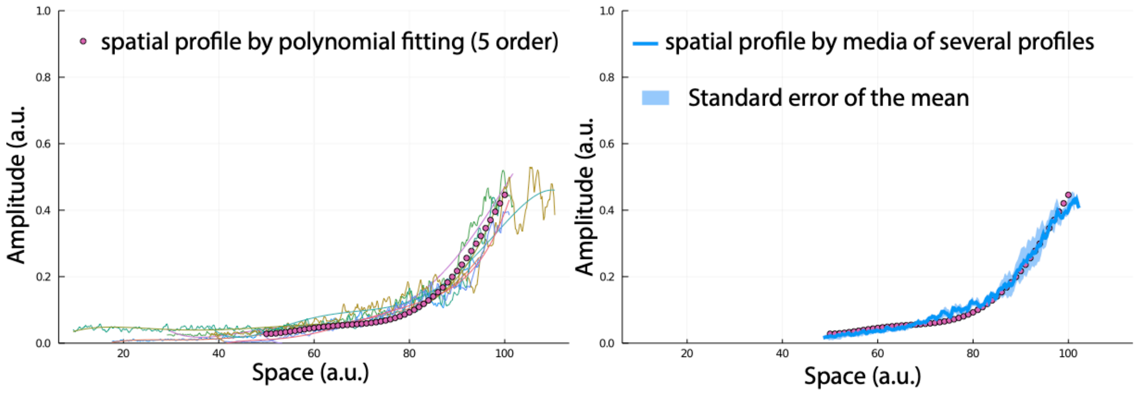

Fig. S1. Example of calculation of average profiles from several experimental measurements. (Left panel) Several profiles are plotted for Hh, together with their corresponding polynomial fitting. Dotted line corresponds to a polynomial plot with parameters calculated from the mean value of all polynomial fittings of the individual curves. (Right panel) Comparison between polynomial average (dots) and the direct average (blue line) over 5 curves, showing a good agreement. Blue ribbon corresponds to the standard error of the mean, showing a small deviation that under-represents the variability in the profiles (this is why a 50% confidence interval is used instead).

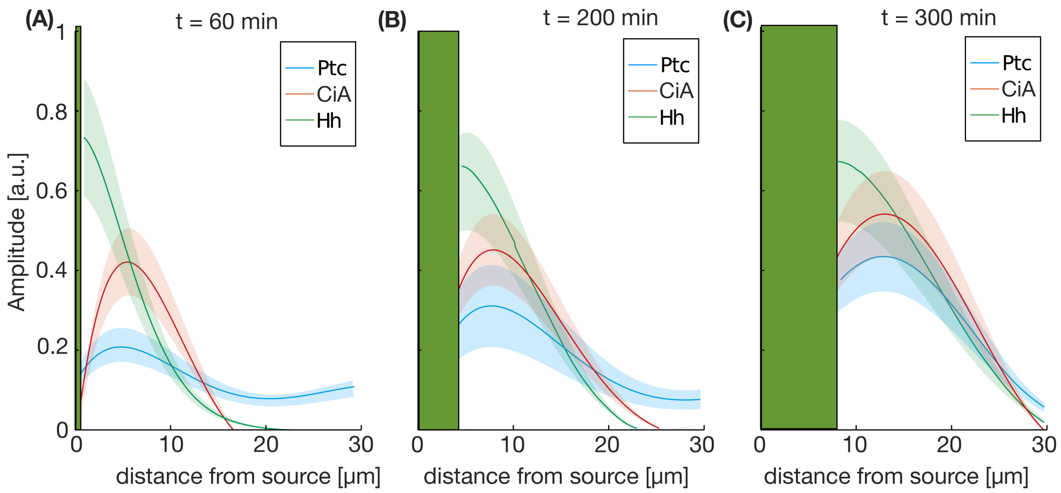

Fig. S2. Spatial profiles of the simulations with a moving boundary at different time points, showing the overlapping of the Ptc and CiA peaks, similarly to the signature profiles of the Compound Eye.

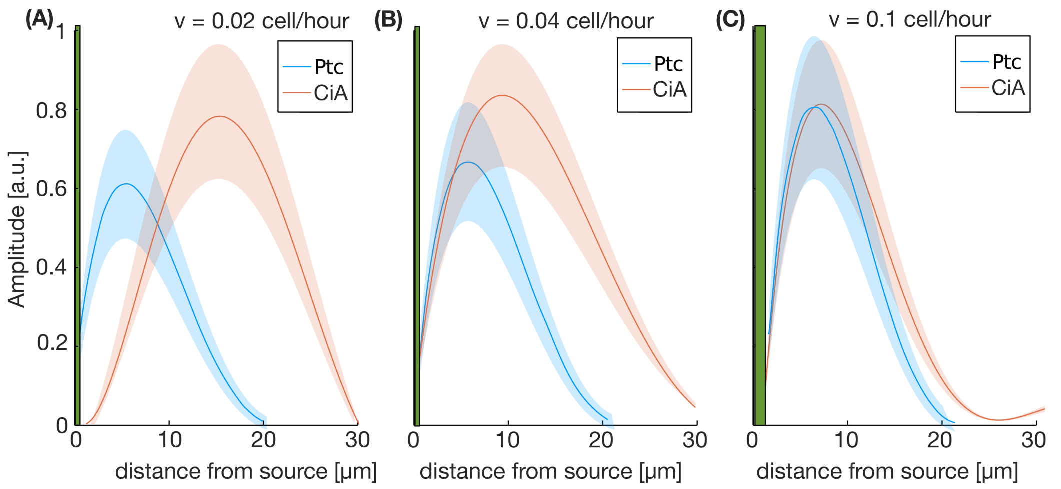

Fig. S3. Profiles of total Ptc (blue) and CiA (red) levels at t =300 min for different velocities of the Hh boundary (moving from left to right at constant speed). This analysis shows that, for the parameter values used, only speeds below 0.04 cell diameters/hour result in a clear peak separation, typical of the signature profile of antenna, ocellus and wing (static Hh sources). Velocity values around 0.04 cell diameters/hour (panel B) show an intermediate situation similar to our experimental results of mutant eye condition (*ey>ato-RNAi;* Figure 4A in main text, central panel). Values above this threshold result in a distribution of peaks that resembles the CE signature profile (moving source).

Development • Supplementary information

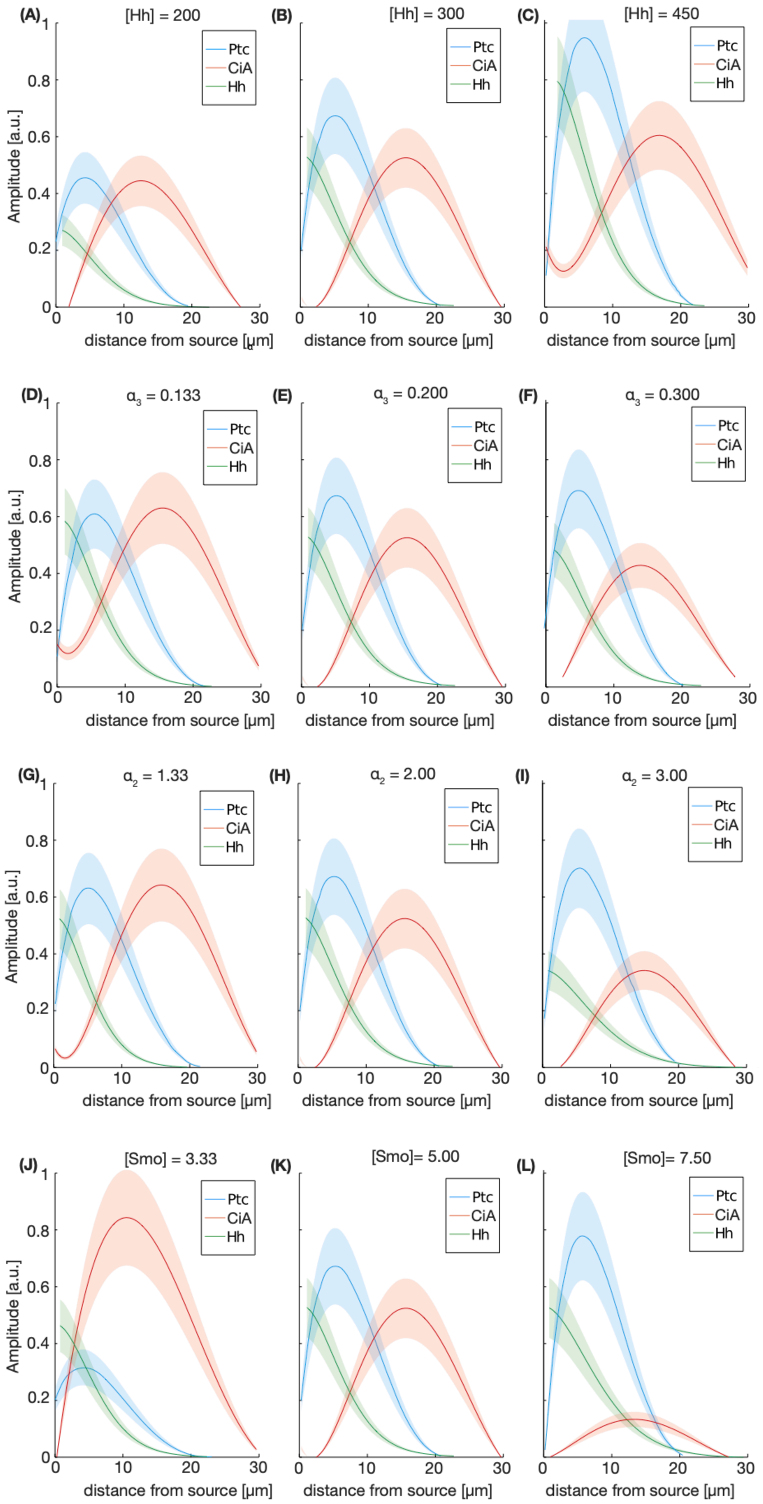

Fig. S4. (A-C) Profiles of Hh (green), Ptc (blue) and CiA (red) for boundary levels of Hh 50% below (A) and above (C) the value used in our simulations. (D-F) Profiles for production rates of Ptc 50% below (D) and above (F) the value used in our simulations. (G-I) Profiles for activation of CiA 50% below (G) and above (I) the value used in our simulations. (J-L) Profiles for levels of Smo 50% below (J) and above (L) the value used in our simulations. This analysis shows that the feature of peak separation in the static signature profile is robust against changes in the key parameters of the model.

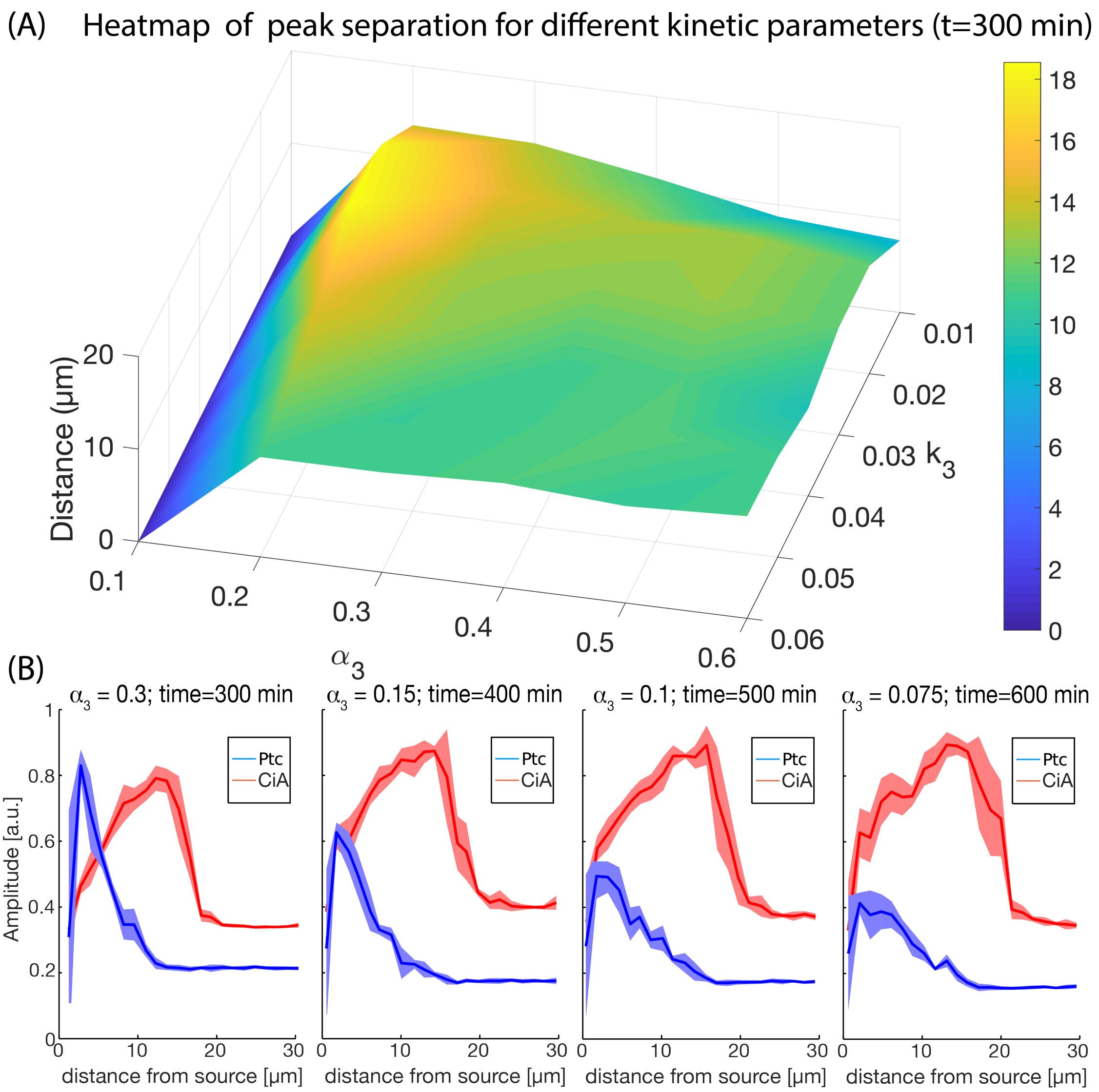

Fig. S5. (A) 3D Heatmap of distance between Ptc and CiA peaks at 300 minutes as a function of the parameters k3 and α3, showing larger distance for slower dynamics of the feedback. (B) Ptc (blue) and CiA (red) profiles for a single simulation with progressively smaller CiA production rate, α3, showing that peak separation occurs later in time as we slow down the rate of CiA production α3 (peak separation increases).

Table S1. Quantification of all experimental profiles used and residuals of all fitting of the experimental results analyzed.

Click here to download Table S1

Supplementary data file 1. MATLAB script to run the numerical mode.

Click here to download data file 1

Development • Supplementary information

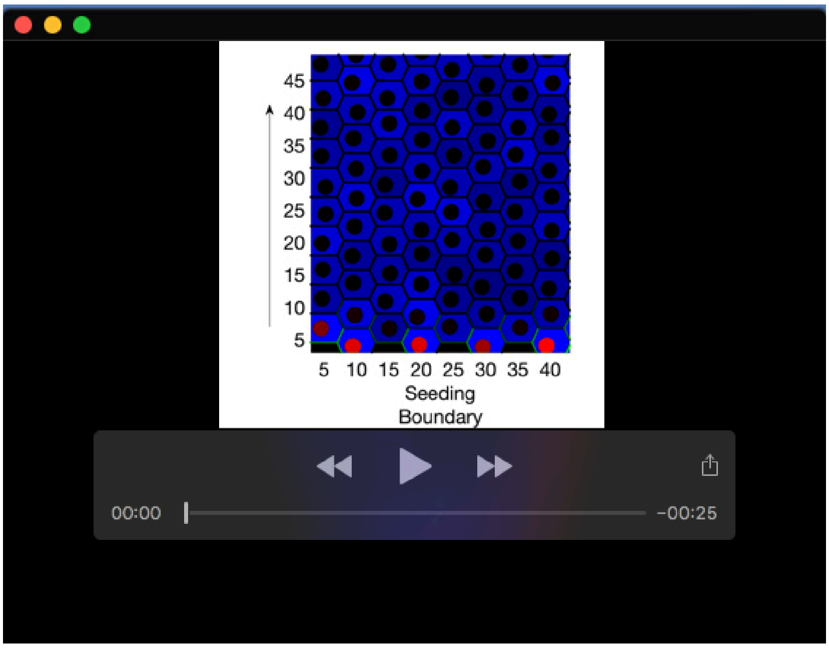

Movie 1. Simulation of the evolution of Hh signaling across a field of cells when the Hh source (bottom) is static. Hh (green), CiA (red, nuclear) and Ptc (blue). Companion to Figure 3.

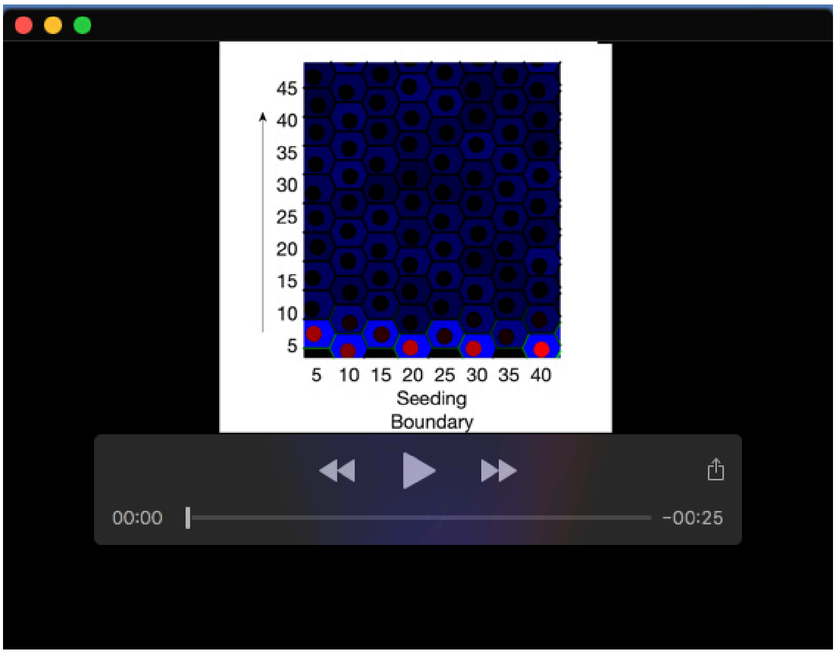

Movie 2. Simulation of the evolution of Hh signaling across a field of cells when the Hh source (bottom) is moving (upwards). Hh (green), CiA (red, nuclear) and Ptc (blue). Companion to Figure 4.

Development • Supplementary information
