# Supplementary material for: The effects of Hh morphogen source movement on signaling dynamics
Source: Development. 2022 Dec 7;149(23):dev199842. doi: 10.1242/dev.199842 (PMC10114110; doi:10.1242/dev.199842)
Supplement: Supplementary file 1 [file develop-149-199842_review_history.pdf]

The effects of Hh morphogen source movement on signaling dynamics

David G. Miguez, Antonella Iannini, Diana Garcia-Morales and Fernando Casares

DOI: 10.1242/dev.199842

Editor: James Briscoe

Review timeline

Original submission:	28 May 2021
Editorial decision:	12 July 2021
First revision received:	23 September 2022
Editorial decision:	24 October 2022
Second revision received:	2 November 2022
Accepted:	2 November 2022

Original submission

First decision letter

MS ID#: DEVELOP/2021/199842

MS TITLE: Patterning on the move: the effects of Hh morphogen source movement on signaling dynamics

AUTHORS: David G Miguez, Antonella Iannini, Diana Garcia-Morales, and Fernando Casares

Dear Dr. Casares,

I have now received all the referees' reports on the above manuscript, and have reached a decision. The referees' comments are appended below, or you can access them online: please go to BenchPress and click on the 'Manuscripts with Decisions' queue in the Author Area.

As you will see, the referees express considerable interest in your work, but have some significant criticisms and recommend a substantial revision of your manuscript before we can consider publication. Both Referee 2 and 3 have a number of related concerns and criticisms that lead them to question whether the conclusion you reach is the only possible explanation for your observations. More exploration of parameter space and/or experiments that measure key parameters and the time scale of the signaling dynamics would strengthen your argument and help address these concerns. Referee 3 suggests alternative explanations that you should consider and either exclude or include in your analysis. In addition, Referee 3 highlights apparent shortcomings in some of the experiments that should be addressed. Each of the referees also seek clarifications on several additional issues.

If you are able to revise the manuscript along the lines suggested, which may involve further experiments, I will be happy to receive a revised version of the manuscript. Your revised paper will be re-reviewed by one or more of the original referees, and acceptance of your manuscript will depend on your addressing satisfactorily the reviewers' major concerns. Please also note that Development will normally permit only one round of major revision.

© 2022. Published by The Company of Biologists under the terms of the Creative Commons Attribution License (https://creativecommons.org/licenses/by/4.0/).

We are aware that you may be experiencing disruption to the normal running of your lab that make experimental revisions challenging. If it would be helpful, we encourage you to contact us to discuss your revision in greater detail. Please send us a point-by-point response indicating where you are able to address concerns raised (either experimentally or by changes to the text) and where you will not be able to do so within the normal timeframe of a revision. We will then provide further guidance. Please also note that we are happy to extend revision timeframes as necessary.

Please attend to all of the reviewers' comments and ensure that you clearly highlight all changes made in the revised manuscript. Please avoid using 'Tracked changes' in Word files as these are lost in PDF conversion. I should be grateful if you would also provide a point-by-point response detailing how you have dealt with the points raised by the reviewers in the 'Response to Reviewers' box. If you do not agree with any of their criticisms or suggestions please explain clearly why this is so.

Reviewer 1

Advance summary and potential significance to field

Understanding the mechanisms of morphogen interpretation is key to elucidate morphogen function in several contexts (patterning and growth in embryonic development, homeostasis, etc).
This manuscripts focuses on Hedgehog (Hh) signalling in the context of Drosophila development.
The authors observe that spatial profiles of Hh signalling species in the compound eye (CE) is crucially different from those in other developing organs --antenna (A), ocelli (OC) and wing primordium (W).
These observations are ascribed to the different dynamics of Hh signal in CE vs A/OC/W, rather than to context-dependent mechanisms, that is to the fact that the source of Hh moves towards receiving cells in CE and is static in the other tissues examined.
The authors support their hypothesis by simulating an ODE model of Hh signalling, as well as by performing an in-vivo perturbation experiment in the CE, wherein the static-source signalling dynamics observed in the A/OC/W is partially recovered.

The manuscript elaborates on the important point that the temporal aspect of signalling is crucial in development.
In particular, it provides an interesting insight into the flexibility of Hh signalling pathway in performing different functions depending on the signal dynamics: establishing several domains of gene expression with a static source, or induce a single cellular fate across entire tissues with a moving source.

The manuscript is well written and the content clearly exposed and, to my knowledge, the content is novel.
I would therefore recommend publication in Development, provided the points below are addressed by the authors.

Comments for the author

1. P.2, "In this organ, once the first photoreceptor..."
What is inducing this first differentiation? Can authors comment on this?

2. Fig.2
What is the time of the snapshots presented?
The central point of the manuscript is that the spatial profile of Ptc/CiA in the CE corresponds ti that of A/OC/W at an early time point: are there quantifications available for this, which can be included in this figure?

3. P.5.
I don't think the differences between the profiles in the different tissues are clearly explained here.
- "The observation of non-coincident peaks...": adaptation of the Hh pathway via the negative feedback loop through Ptc is known, hence this should not be necessarily surprising.

© 2022. Published by The Company of Biologists under the terms of the Creative Commons Attribution License (https://creativecommons.org/licenses/by/4.0/).

- "For example, high Ptc...": Is this comparison made between quantities close to the source, or between max values? I do not see how the Ptc/CiA ratio is overall different between A/OC and W from Fig.2B. Can the authors clarify the differences they are highlighting?

4. P.6.
The first hypothesis (core pathway modified in an organ-specific manner) is not explored. What would such hypothesis mean mechanistically? Is it, e.g. a context-dependent degradation rate of signalling species? Are there data in the literature suggesting or excluding this hypothesis? Can the authors elaborate on this?

5. Model.
- Smoothened does not degrade in the model. Is this a fair assumption?
- Please, mention in the main text that the subscript "i" refers to the cells. Also, even though it is probably not crucial, could the authors mention in the methods how the Hh_i is calculated from the off-lattice Hh --is it the value at the centre of the cell?

6. P.8. "If the receiving cells, as a result of the differentiation wave, became a Hh source faster than ..."
Here the authors describe the effect of the Hh positive feedback loop, in a certain regime of time-scale separation. I understand that the production of Hh would need to be controlled by the Hh signalling intracellular effector CiA, in order to close the positive feedback loop. The authors mention that the proneural gene atonal "triggers a series of regulatory steps that lead ultimately to the differentiation of new R-cells", which entails production of Hh. This makes me wonder whether the steps leading to production of Hh in ato+ cells are indeed much faster than the production of Ptc by CiA. If this is known, can the authors comment on this?

7. Fig.4
Can the profiles from the simulations at different time points be shown here or in a supplementary figure?

Minor suggestions

- For better readability, I would suggest to use the "[...]" notation for all the concentrations, e.g. [Hh], [Hh Ptc], etc.

- P.7: "The binging of a diffusible..". Correct to "binding".

- P.7: "Hh is produce at rate..". Correct to "produced".

- P.8: "...which are responsible of the...". Correct to "responsible for".

- Nomenclature for model parameters.
- The parameter m, referred to as "Hill exponent" is normally called "Hill coefficient".
- The concentration parameter M, referred to as "Hill coefficient" may be referred to as "concentration threshold". As the Hill coefficient is relatively high (m = 5), I would refer to it as "threshold" in this case. For instance, M2 would be "Smo threshold for Ci production", and M3 would be "CiA threshold for Ptc repression".
- I would call the parameters alpha_2 and alpha_3 "maximum production rate"

- The value of alpha_1, seems very high, if the units are the same as in the plots reported in the main text (where I would expect to have alpha_1 ~ 1).

<u>Reviewer 2</u>

Advance summary and potential significance to field

In this article Miguez et al explore the signaling dynamics of Hh in four different organs in the developing fly (the compound eye (CE), antenna, ocellus and wing), and ask whether organ specific modifications in the Hh signaling pathway need to be invoked to understand how Hh mediates diverse patterning. The authors visualize the key signaling players: Ptc, Ci and Hh in the four organs

© 2022. Published by The Company of Biologists under the terms of the Creative Commons Attribution License (https://creativecommons.org/licenses/by/4.0/).

(Fig. 2) and find that the antenna, ocellus and wing show the same qualitative profiles whereas the signaling profiles in the CE are different. In particular the peak signaling levels of Ptc and Ci coincide in space in the CE but not the other three organs. The authors then wondered what is the source of this discrepancy and develop a theoretical framework to model Hh dynamics. This model assumes a static source and the authors look at the signaling dynamics at different time points and deduce that the differences they observe experimentally may reflect the fact that the signaling pathway is not at steady state in the CE.

They hypothesize that the moving source of Hh in the CE could explain this departure from steady state when the timescale of the source speed is faster than the timescale that defines the Hh pathway's signaling dynamics. They predict that slowing the source down in the CE would lead to peaks of Ptc and Ci signaling that are further apart from one another as seen in the other organs. The authors then test this prediction experimentally using ato-RNAi flies where the Hh source movement is impacted. In these conditions they find that the signaling dynamics do shift although the peak signaling intensities of Ptc and Ci do not become separated as expected. The authors then conclude that the two signaling profiles they observed initially (in the CE vs the other three organs) arise from the same signaling network but differ qualitatively due to the source movement.

Comments for the author

In this article Miguez et al explore the signaling dynamics of Hh in four different organs in the developing fly (the compound eye (CE), antenna, ocellus and wing), and ask whether organ specific modifications in the Hh signaling pathway need to be invoked to understand how Hh mediates diverse patterning. The authors visualize the key signaling players: Ptc, Ci and Hh in the four organs (Fig. 2) and find that the antenna, ocellus and wing show the same qualitative profiles whereas the signaling profiles in the CE are different. In particular, the peak signaling levels of Ptc and Ci coincide in space in the CE but not the other three organs. The authors then wondered what is the source of this discrepancy and develop a theoretical framework to model Hh dynamics. This model assumes a static source and the authors look at the signaling dynamics at different time points and deduce that the differences they observe experimentally may reflect the fact that the signaling pathway is not at steady state in the CE. They hypothesize that the moving source of Hh in the CE could explain this departure from steady state when the timescale of the source speed is faster than the timescale that defines the Hh pathway's signaling dynamics. They predict that slowing the source down in the CE would lead to peaks of Ptc and Ci signaling that are further apart from one another as seen in the other organs. The authors then test this prediction experimentally using ato-RNAi flies where the Hh source movement is impacted. In these conditions they find that the signaling dynamics do shift although the peak signaling intensities of Ptc and Ci do not become separated as expected. The authors then conclude that the two signaling profiles they observed initially (in the CE vs the other three organs) arise from the same signaling network but differ qualitatively due to the source movement.

The article is well written and easy to follow and the authors make some interesting observations. I do have several concerns and suggestions for improvement that I list below.

My first concern is conceptual. I have to admit I have missed what the novelty of the study really is. Although the authors discuss the idea that Hh operates in two different modes, a "spatial" and a "mobile" mode in the antenna, ocellus and wing versus the CE (last paragraph, page 10) I do not see how this is an insight that comes from the current study. It was already known that Hh operates as a morphogen in antenna, ocellus and wing and a traveling wave in the CE (the authors discuss this in the introduction, page 2). As far as I can tell the new insight from this study comes from establishing that the signaling dynamics in the CE are not in steady state and proposing that the movement of the source may have to do with this lack of stationarity. Perhaps I have missed something but I think this is an important point to clarify conceptually.

A second major concern is the way the model is employed in this study. I list my concerns below:

1. The model assumes 14 parameters (listed on Table 1) and the values these parameters take are important as they determine how fast the signaling dynamics are relative to the source movement. I found the arguments the authors use for the parameters they chose not convincing.

© 2022. Published by The Company of Biologists under the terms of the Creative Commons Attribution License (https://creativecommons.org/licenses/by/4.0/).

a. The diffusion coefficient of Hh D should follow D = \lambda^2 * k where \lambda is the decay length of Hh is and k = (1/60)mins (13um according to a study the authors cite). Is this right? This does not give a D of 0.1um^2/min. I would suggest writing down the calculations the authors have performed to get the diffusion coefficient to avoid confusion.
b. How the authors have estimated binding and unbinding rates of Hh to its receptor is also not clear. They make a brief mention of affinities but they don't explain how the rates were inferred from the affinities. Can you please clarify?
c. Importantly, I do not see how the degradation rates for Hh, Ptc and the Hh-Ptc complex were estimated. The table simply states "calibrated". What does this mean? These parameters are very important as they determine whether the timescale in which the signaling dynamics reach steady state. I suspect there is no obvious way to obtain these values directly in which case I think a better use of the model is to perform a phase-space analysis to explore the parameter regions in which the authors' conclusions about the significance of the Hh source movement are valid.
d. The Hill coefficient for the feedback function in the signaling dynamics was also "calibrated". What does this mean? Again, this is a parameter that is likely to impact the overall dynamics and steady state as it affects the sharpness of the feedback.
e. How were the Hill coefficients of Ci and Ptc expression estimated? What are the confidences for these values?
f. I can see how some parameters may not be easily quantified and this is ok. I think what is important is that authors clarify how important their choice of parameters is for their conclusions (given that many parameters in the model are free) and explain clearly how parameters values were estimated, calibrated or inferred from the literature.
g. It would be useful to see a comparison of the model and the experiments when the model considers a moving source. Fig. 4 shows a qualitative picture but it would be much more informative if the authors plotted the signaling and ligand dynamics in this case and then repeat the simulations with a progressively slower moving source. Would this lead to the peaks becoming progressively more separated? It would be good to also recapitulate the mutant/RNAi versus control dynamics and quantifications in Fig. 4 with the model using a static, fast and slow moving source with all other parameters kept the same.
h. This is a more minor point but I find the notation used for constructing the model confusing. Instead of using the molecular names as parameters I suggest defining simple letters (e.g. C for concentration, R for receptor, S for signal or something along these lines). It's currently quite hard to read the equations and figure out which letters are indices and which are part of a parameter or variable. Also since only one diffusion coefficient is used the subscript is not necessary for D_1.

2. Relevant to some of the concerns I raised regarding the model and given that the assumption that the source in the CE moves faster than the signaling dynamics, is there an experiment that could provide an estimate for the time scale of the signaling dynamics downstream of Hh? FRAP or pulse-chase come to mind, are these or other experiments that tackle this point feasible?

Another issue is that the Ptc and Ci peaks in the RNAi experiment do not become separated in the experiment as predicted by the model. In fact, the authors only compare the amplitude of Ptc and the decay of Ptc in the two conditions. I guess this could in principle be explained if the dynamics is still not at steady state with a slow-moving source. Can the authors elaborate on this point? Can the speed of the source in the RNAi conditions be quantified and compared to the control?

Some minor points:
- Why were polynomial fits performed to the experimental profiles? Why not show the average of the 5 experimental profiles instead? If the authors insist to perform the fits then more

© 2022. Published by The Company of Biologists under the terms of the Creative Commons Attribution License (https://creativecommons.org/licenses/by/4.0/).

details of the functions fitted, quality of fits and error estimations should be provided in the methods.
- I wonder if it makes more sense to normalize the curves of the dynamics to make them more directly comparable. Are the differences in amplitudes in the experiments meaningful if different FPs are used?
- Can the authors report the p-values for all their statistical tests in the figure legend or methods?
- The ribbons in the experiments shows the sem but in the model they show the 50% CI. Can the authors also show the sem in the model to make the plots comparable?
- Fig. 4E is not easy to interpret. I would show the quantifications instead.
- Fig. 4C, shouldn't the exponent be dimensionless (not in a.u.)?

Reviewer 3

Advance summary and potential significance to field

Hedgehog (Hh) acts as a patterning morphogen in a variety of developmental contexts. A universal feature of Hh signalling is the upregulation of its receptor Ptc and this feedback self-limits its range of action. In this manuscript, the authors investigate the patterns of Hh-GFP, Ptc, and the signalling effector CiA in the Drosophila compound eye (CE), wing, ocelli, and antenna. They found that the spatial peaks of Ptc and CiA differ in the CE with respect to the other tissues examined. Using a mathematical model, the authors observe that an early time point in the dynamics of the signal somehow resemble the patterns in the CE and suggest that this is due to the moving source of Hh, driven by the progression of the morphogenetic furrow (which does not occur in the other organs). The authors then attempt to test this hypothesis experimentally by inhibiting genetically the progression of the morphogenetic furrow.

Comments for the author

In my opinion, the mathematical analysis is not very useful and the conclusions of the manuscript are not necessarily supported by the evidence that is presented (see below). Therefore, I do not support publication of the manuscript in its current form.

1. In Fig. 2 the authors quantify differences in the profiles of Hh-GFP, Ptc, and CiA in the CE, Antenna, Ocellus, and Wing. They focus on the following two qualitative observations of their data: (i) "The signaling profiles of Ptc and CiA in the wing, antenna and ocellus were qualitatively similar, with a first peak of Ptc followed by a peak in CiA further from the Hh source. On the other hand, the signaling signature of Hh in the CE was qualitatively different from the others, with a peak expression of Ptc that is coincident in space with a maximum in CiA". (ii) "the signal intensity of Ptc at this maximum was significantly lower in the CE that in the other tissues."

The authors claim that these findings are counterintuitive and they justify the need to build a mathematical model to make sense of these data, but the reality is that there is little need of the mathematical model since it displays exactly what would be expected by intuition. Because Ptc is a repressor of the system, it is expected that a peak of Ptc near the Hh source will shift the expression of CiA.

In figure 2B of the CE, it is not clear if x=0 corresponds to the position of the morphogenetic furrow (source of Hh-GFP), or how is x=0 defined? From the profile of Hh-GFP it appears that there is a non-zero plateau of Hh expression at least for the first 10 microns. If these cells are producing Hh, then it is expected that Ptc levels are lower in this region since more Hh is binding free Ptc in the system.

2. The authors then solve their mathematical model using parameters from previous studies and observe that at earlier stages of the dynamics the peaks of Ptc and CiA coincide, but at later times these peaks start to separate from one another. This led the authors to propose "the model reproduces the profiles for the CE and the other organs as an early and a later time point, respectively, along its temporal dynamics". This is a major flaw of the paper. First, because the profiles of Fig. 2C (CE) and Fig. 3D do not look that similar as they claim. But the main problem

© 2022. Published by The Company of Biologists under the terms of the Creative Commons Attribution License (https://creativecommons.org/licenses/by/4.0/).

here is that even if we accept that they look similar, there is no evidence that the CE corresponds to an earlier stage of Hh signalling dynamics. There are other plausible explanations of why the peak of CiA does not separate from the peak of Ptc expression in the CE (as I said in the previous point, this is most likely due to cells gradually becoming source of Hh, which reduces the levels of free Ptc although these do not become sufficiently strong to repress the expression of CiA). But there are other explanations that could be considered if they want to use their model as an exploratory tool. For example, from Fig. 2, it appears that the levels of Hh are different in each of the organs; this will also affect the levels of Ptc that will be expressed in receiving cells; having different Hh levels at the source could affect the dynamics without affecting the network.

3. The experiment in Fig. 4 in which the Gal4-UAS system is used to reduce the movement of the morphogenetic furrow shows that in fact the levels of Ptc in receiving cells and the differences in the peaks of Ptc and CiA are related to the movement of the source of Hh, but this may have nothing to do with the dynamic state of the signal (which is the main conclusion of the paper). Once again it may have to do with the levels of free Ptc that ramp up when receiving cells are not allowed to become source cells. Therefore, I do not find that this experiment support the authors' claims.

In addition, this experiment was not done rigorously and the data are not presented adequately. They use different temperatures to control the strength of the Gal4 system, but they should use the Gal80ts to provide a more robust time control of atoRNAi expression. Actually the use of the Gal80ts may be used to assay the patterns of CiA and Ptc in early vs. late signaling. Moreover, the authors do not show any image of the actual data (stained discs) that supports the results of panels B and C. In fact, panels B and C are unnecessary because the information can be easily obtained from A.

In summary, the differences in the spatial distributions of the peaks of Ptc and CiA in the CE with respect to the other tissues are not necessarily related to an earlier dynamic state of the signalling.

4. In the discussion, the authors state that their data supports that in the wing/antenna/ocellus, Hh signalling works as a traditional morphogen, whereas in the CE it does not. However, by looking at the profiles it seems to me that this is quite the opposite. In fact, it is in the CE that the CiA profile (which is the transcriptional effector of the signal) looks more graded (see Fig. 2B). Actually, the fact that there is a stronger peak of Ptc in the wing/antenna/ocellus is more against the traditional morphogen hypothesis than in the CE. (In fact, a previous study in the wing by Nahmad and Stathopoulos (2009) that the authors cite and other papers by Briscoe and colleagues in the vertebrate neural tube do not support that Hh is a traditional morphogen).

Other comments:

- In the introduction, some key references are not cited. For example, the idea that Hh-dependent ptc upregulation self-limits the range of the Hh gradient in Drosophila was originally proposed by Chen and Struhl, 1996.

- The model uses a Hill function for the activation of CiA, even though this is not a transcriptional activation process. How does this choice affect the results? Also, the authors used the parameter values of M3=5 and M2=2.5. Therefore, it is not surprising that the peak of CiA extends beyond the one for Ptc as more signal is required to activate Ptc than CiA. What is the evidence of this choice in the parameters and how does the dynamics change in other parametric conditions?

- In page 10, the authors say that "Figure 4B,C shows three snapshots of this scenario taken at three different time points". Should say 4E.

- It is somehow redundant to put the Hh network twice in Fig. 1F and Fig. 3A.

- Use nomenclature consistently, in Fig. 3A replace Cia for CiA.

© 2022. Published by The Company of Biologists under the terms of the Creative Commons Attribution License (https://creativecommons.org/licenses/by/4.0/).

First revision

Author response to reviewers' comments

First of all, we would like to thank all three reviewers for their detailed and insightful revisions of our paper, which have been very useful. Second, we would also like to apologize for having taken such a long time to resubmit the revised manuscript. While the new computational analyses were finished in a week after having received the reviews, the experiment that finally goes in the new Figure 4 and which, after hearing the referees, seemed to us as important too, took us forever. We realized that some of the stocks we thought we had in our stock collection had been lost during the lockdown and the slow reactivation that followed. Then, stocks sent by colleagues either did not work as should have, or the experimental designed was somehow flawed. Finally, after many attempts at an experiment that would allow us to follow in time the evolution of the Hh-induced signaling profiles, we were successful. In what follows, we answer one by one all the points raised by each of the referees.

Reviewer 1 Advance Summary and Potential Significance to Field:
Understanding the mechanisms of morphogen interpretation is key to elucidate morphogen function in several contexts (patterning and growth in embryonic development, homeostasis, etc). This manuscript focuses on Hedgehog (Hh) signalling in the context of Drosophila development. The authors observe that spatial profiles of Hh signalling species in the compound eye (CE) is crucially different from those in other developing organs --antenna (A), ocelli (OC) and wing primordium (W). These observations are ascribed to the different dynamics of Hh signal in CE vs A/OC/W, rather than to context-dependent mechanisms, that is to the fact that the source of Hh moves towards receiving cells in CE and is static in the other tissues examined. The authors support their hypothesis by simulating an ODE model of Hh signalling, as well as by performing an in-vivo perturbation experiment in the CE, wherein the static-source signalling dynamics observed in the A/OC/W is partially recovered.

The manuscript elaborates on the important point that the temporal aspect of signalling is crucial in development. In particular, it provides an interesting insight into the flexibility of Hh signalling pathway in performing different functions depending on the signal dynamics: establishing several domains of gene expression with a static source, or induce a single cellular fate across entire tissues with a moving source.

The manuscript is well written and the content clearly exposed and, to my knowledge, the content is novel.
I would therefore recommend publication in Development, provided the points below are addressed by the authors.

Reviewer 1 Comments for the Author:
1. P.2, "In this organ, once the first photoreceptor..."
What is inducing this first differentiation? Can authors comment on this?
R1. PR differentiation is triggered by the BMP2 molecule Dpp, produced by the cells surrounding the eye primordium (and which will become the posterior head capsule). We have now introduced this description in the text.

2. Fig.2 What is the time of the snapshots presented? The central point of the manuscript is that the spatial profile of Ptc/CiA in the CE corresponds to that of A/OC/W at an early time point: are there quantifications available for this, which can be included in this figure?
R2. This figure includes snapshots of late L3 discs (we specify this point in the figure legend now). During L3, while the MF moves across the disc, we have not observed qualitative change in the relative profiles of Ptc and CiA (that is, the peaks overlap throughout). For the other discs, we have not surveyed earlier time points. But, since the AP boundary, at least for the wing, is established much earlier (actually in the embryo), we would expect the dynamic displacement of the Ptc and CiA peaks to have occurred already in L1. We checked in early L3 wing discs, though, following your comment and, indeed, the peak displacement is observable already. See attached figure:

© 2022. Published by The Company of Biologists under the terms of the Creative Commons Attribution License (https://creativecommons.org/licenses/by/4.0/).

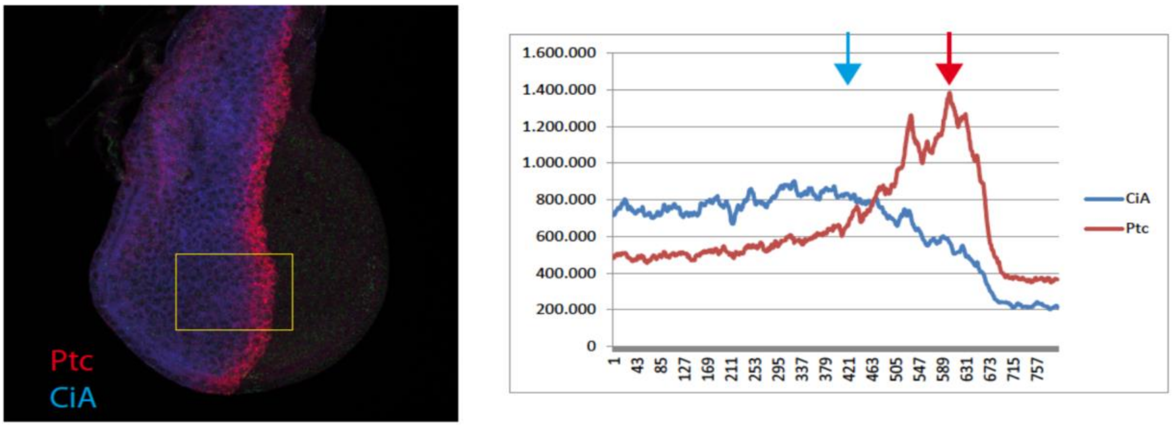

Left: confocal micrograph of an early-mid L3 wing disc stained for Ptc and CiA (same stainings as reported in the paper). Right: quantitative profiles from the boxed region (left). Signal maxima (marked approximately by the arrows) are already displaced at this stage.

3. P.5.
I don't think the differences between the profiles in the different tissues are clearly explained here. - "The observation of non-coincident peaks...": adaptation of the Hh pathway via the negative feedback loop through Ptc is known, hence this should not be necessarily surprising.
R3.1. The referee suggests that the fact that the peaks of Ptc and CiA separate dynamically is not totally surprising in a system with a negative feedback. We have to admit that this intuition was not obvious to us when we started this work, especially because this is a spatially extended system where the spatial response of the signaling components (Ptc and CiA) depends on a Hh gradient that is dynamically modified by the signal (through Ptc). Actually, the separation of the peaks is observed in our computational model only for a subset of parameters. In any case, though, we have posed the problem we study more clearly by modifying the sentence as "The observation of non-coincident peaks for CiA and Ptc in wing, antenna and ocellus might not have been surprising in a spatially extended system with Ptc exerting a negative feedback, but why is it not observed in the CE?".

- "For example, high Ptc...": Is this comparison made between quantities close to the source, or between max values? I do not see how the Ptc/CiA ratio is overall different between A/OC and W from Fig.2B. Can the authors clarify the differences they are highlighting?
R3.2 In this sentence, we are referring to the maximum levels of Ptc, but based on the comment of the referee, we decided to omit the description of the relative peak expression of Ptc and CiA to avoid blurring the main observations.

4. P.6.
The first hypothesis (core pathway modified in an organ-specific manner) is not explored. What would such hypothesis mean mechanistically? Is it, e.g. a context- dependent degradation rate of signalling species? Are there data in the literature suggesting or excluding this hypothesis? Can the authors elaborate on this?
R4. We have left the discussion of the organ-specific modifications of the pathway to the end of the paper. Here we describe results (from one of our groups) showing how the nuclear factor Dachshund (Dac) helps stabilizing CiA in the CE. By doing so, Dac would accelerate the dynamics of the systems, promoting the separation of Ptc and CiA maxima -contrary to what is observed. We have included this discussion in the revised version of the paper.

5. Model.
- Smoothened does not degrade in the model. Is this a fair assumption?
R5.1. The way in which Ptc inhibits Smo activity is not totally clear yet, but studies of the vertebrate homologues, PTCH1 and SMO, suggest that this regulation might involve Ptc reducing the availability of accessible cholesterol to Smo (by acting as a cholesterol transporter), although evidence in *Drosophila* is still lacking (reviewed in Radhakrishnan 2020: 10.1038/s41589-020-00678-2). We have decided to simplify this interaction, likely mediated by cholesterol, formulating it as an inhibitory interaction. This has been the first simplification,

© 2022. Published by The Company of Biologists under the terms of the Creative Commons Attribution License (https://creativecommons.org/licenses/by/4.0/).

although it has been shown that Ptc and Smo are part of a large multiprotein complex (Walthall 2007; 10.1242/jcs.03382) and indeed Ptc has been shown to downregulate Smo protein in wing discs post-transcriptionally (Denef 2000; 10.1016/S0092-8674(00)00056-8), two pieces of evidence supporting our formulation of the interaction between Ptc and Smo as a(n inhibitory) complex. The second simplification is the one noted by the reviewer. In it, we assume that Smo is in a state of dynamic equilibrium in which production and degradation are equal, canceling each other (and constant - that is, degradation does not depend on Ptc concentration, for example). Since the mechanisms are not clear, we decided to adopt this simplification since otherwise, the mounting in parameter numbers would make the interpretation of the model more cumbersome. In this form, we think it captures the core effect of the feedback regulation. We have introduced a brief note in the model section to bring the attention of the reader to this simplification.

- Please, mention in the main text that the subscript "i" refers to the cells. Also, even though it is probably not crucial, could the authors mention in the methods how the Hh_i is calculated from the off-lattice Hh --is it the value at the centre of the cell?
R5.2 We apologize because we didn't mention that the sub index i corresponds to the processes in each individual cell. For simplicity and to improve the clarity of the equations, we removed the sub-index i from the equations, and explicitly say that the first equation is solved globally, while the rest of them are solved for each cell individually.

In addition, following your suggestion, we added an explanation of how Hh is calculated for each cell (the average value over the cell area). We also added brackets for the concentrations (suggested by this same referee). We hope that these changes result in a simpler and clearer set of equations.

6. P.8. "If the receiving cells, as a result of the differentiation wave, became a Hh source faster than ..."
Here the authors describe the effect of the Hh positive feedback loop, in a certain regime of time-scale separation. I understand that the production of Hh would need to be controlled by the Hh signalling intracellular effector CiA, in order to close the positive feedback loop. The authors mention that the proneural gene atonal "triggers a series of regulatory steps that lead ultimately to the differentiation of new R-cells", which entails production of Hh. This makes me wonder whether the steps leading to production of Hh in ato+ cells are indeed much faster than the production of Ptc by CiA. If this is known, can the authors comment on this?

R6. This is a very interesting point, but one about which we can only speculate, because, to our knowledge, no one has measured the rates involved. Before anything, the closing of the loop is as the reviewer suggests: Hh→ CiA→ Ato→ (PR cell identity)→ Hh, which means that it closes indirectly, with several steps interposed. At 25°C, the differentiation wave advances roughly one cell diameter per hour (that is, everything necessary for the induction of R cells and their production of signals, including Hh, happens at that pace). In our dynamical system, the signal needs to first activate CiA (biochemical cascade), this is then followed by an increase in Ptc (transcriptional/translational process) and then finally a downregulation of CiA (biochemical cascade) occurs. At least, transcription and translation happens on the scale of minutes. And even if generally biochemical events might happen much faster (seconds), it is possible that some of the biochemical processes involved in Hh signal transduction require subcellular relocalizations, which might be more sluggish. So, very roughly, it might be that the timing imposed by the pace of retina differentiation (1 hour) is, indeed, tight for the loop to be closed. In some additional experiments carried out for the revision of this paper, we have induced the expression of Hh ectopically in the wing pouch. Although the time resolution of the process afforded by this experiment is not great, the fact that we find discs in which the Hh expression is very clear and yet the separation of the CiA and Ptc peaks have not occurred (something that does happen in this experiments in later time points) suggests to us that the “peak displacement” of Ptc and CiA maxima is not fast (see new Figure 4).

7. Fig.4
Can the profiles from the simulations at different time points be shown here or in a supplementary figure?

© 2022. Published by The Company of Biologists under the terms of the Creative Commons Attribution License (https://creativecommons.org/licenses/by/4.0/).

R7. Following the suggestion from reviewer #1, also shared by reviewer #3 we generated a new supplementary figure where we quantified the profiles at the same three different time points. These profiles make the comparison with the profiles when the boundary is not moving (Fig 3 D-F) more direct, so we thank both referees for this useful suggestion.

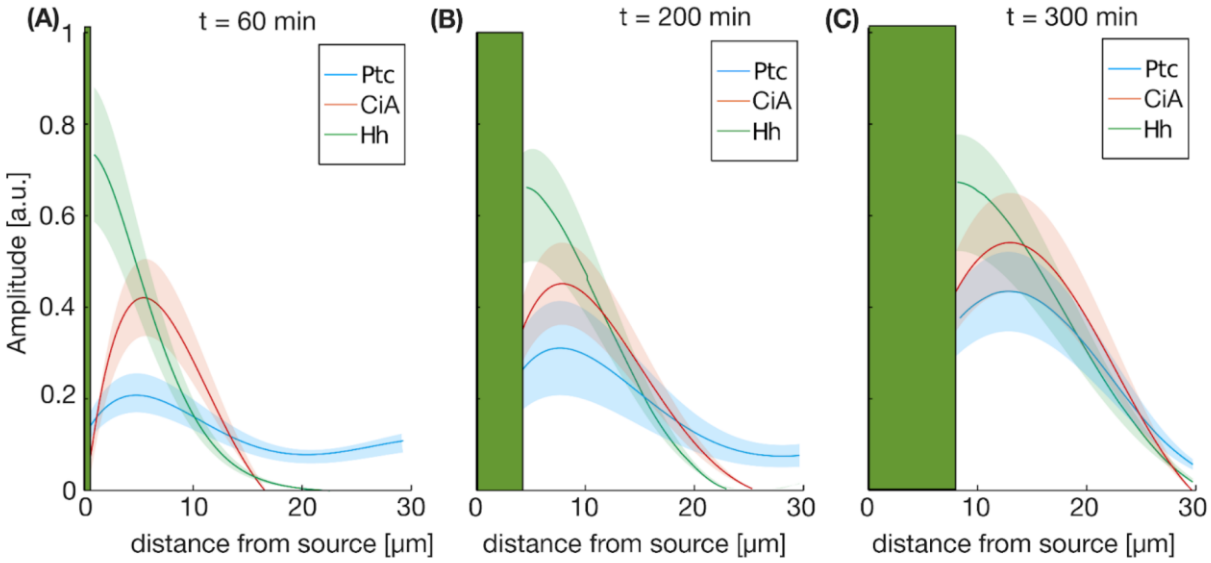

Supp. Figure 2, legend. Spatial profiles of the simulations with a moving boundary at different time points, showing the overlapping of the Ptc and CiA peaks, similarly to the signature profiles of the Compound Eye.

Minor suggestions

- For better readability, I would suggest to use the "[...]" notation for all the concentrations, e.g. [Hh], [Hh Ptc], etc.

Thank you for this suggestion. We tried the brackets notation and the equations are more readable this way. It also allows us to simplify the notation by removing all the dots that indicate multiplication. We also simplified the equations removing the “i” subindexes (based on one of your previous suggestions).

- P.7: "The binging of a diffusible..". Correct to "binding".
Amended.

- P.7: "Hh is produce at rate..". Correct to "produced".
Amended.

- P.8: "...which are responsible of the...". Correct to "responsible for".
Amended.

Thank you for your careful reading of the manuscript. These and other typos have been corrected.

- Nomenclature for model parameters.
- The parameter m, referred to as "Hill exponent" is normally called "Hill coefficient".
- The concentration parameter M, referred to as "Hill coefficient" may be referred to as "concentration threshold". As the Hill coefficient is relatively high (m = 5), I would refer to it as "threshold" in this case. For instance, M2 would be "Smo threshold for Ci production", and M3 would be "CiA threshold for Ptc repression".
- I would call the parameters alpha_2 and alpha_3 "maximum production rate"

Thank you again. We have followed your advice and changed the nomenclature for M, m and alpha.

© 2022. Published by The Company of Biologists under the terms of the Creative Commons Attribution License (https://creativecommons.org/licenses/by/4.0/).

- The value of alpha_1, seems very high, if the units are the same as in the plots reported in the main text (where I would expect to have alpha_1 ~ 1).

We apologize for the confusion; alpha_1 represents concentration, while alpha_2 and alpha_3 represent rates (concentration /time). In fact, alpha_1 represents the value set as a boundary condition in the lower boundary of the system. To avoid this confusion and to simplify the equations, the boundary level of Hh is not defined now as alpha_1. Instead, we mention the value at the boundary in the Methods section and in table 1.

$$\frac{\partial[Hh]}{\partial t} = D\frac{\partial^2[Hh]}{\partial \vec{r}^2}$$

$$\frac{\partial[Hh]}{\partial t} = k_2[HhPtc] - k_1[Hh][Ptc] - \beta_1[Hh]$$

$$\frac{\partial[HhPtc]}{\partial t} = k_1[Hh][Ptc] - k_2[HhPtc] - \beta_3[HhPtc]$$

$$\frac{\partial[Ptc]}{\partial t} = -k_1[Hh][Ptc] + k_2[HhPtc] - k_3[Smo][Ptc] + k_4[SmoPtc] - \beta_3[Ptc] + \frac{\alpha_3[CiA]^m}{[CiA]^m + M_3^m}$$

$$\frac{\partial[Smo]}{\partial t} = -k_3[Smo][Ptc] + k_4[SmoPtc]$$

$$\frac{\partial[SmoPtc]}{\partial t} = k_3[Smo][Ptc] - k_4[SmoPtc]$$

$$\frac{\partial[CiA]}{\partial t} = \frac{\alpha_2[Smo]^m}{[Smo]^m + M_2^m} - \beta_2[CiA]$$

Reviewer 2 Comments for the Author:
In this article Miguez et al explore the signaling dynamics of Hh in four different organs in the developing fly (the compound eye (CE), antenna, ocellus and wing), and ask whether organ specific modifications in the Hh signaling pathway need to be invoked to understand how Hh mediates diverse patterning. The authors visualize the key signaling players: Ptc, Ci and Hh in the four organs (Fig. 2) and find that the antenna, ocellus and wing show the same qualitative profiles whereas the signaling profiles in the CE are different. In particular, the peak signaling levels of Ptc and Ci coincide in space in the CE but not the other three organs. The authors then wondered what is the source of this discrepancy and developed a theoretical framework to model Hh dynamics. This model assumes a static source and the authors look at the signaling dynamics at different time points and deduce that the differences they observe experimentally may reflect the fact that the signaling pathway is not at steady state in the CE. They hypothesize that the moving source of Hh in the CE could explain this departure from steady state when the timescale of the source speed is faster than the timescale that defines the Hh pathway's signaling dynamics. They predict that slowing the source down in the CE would lead to peaks of Ptc and Ci signaling that are further apart from one another as seen in the other organs. The authors then test this prediction experimentally using ato-RNAi flies where the Hh source movement is impacted. In these conditions they find that the signaling dynamics do shift although the peak signaling intensities of Ptc and Ci do not become separated as expected. The authors then conclude that the two signaling profiles they observed initially (in the CE vs the other three organs) arise from the same signaling network but differ qualitatively due to the source movement.

The article is well written and easy to follow and the authors make some interesting observations. I do have several concerns and suggestions for improvement that I list below.

1- My first concern is conceptual. I have to admit I have missed what the novelty of the study really is. Although the authors discuss the idea that Hh operates in two different modes, a "spatial" and a "mobile" mode in the antenna, ocellus and wing versus the CE (last paragraph, page 10) I do not see how this is an insight that comes from the current study. It was already known that Hh operates as a morphogen in antenna, ocellus and wing and a traveling wave in the CE (the authors discuss this in the introduction, page 2). As far as I can tell the new

© 2022. Published by The Company of Biologists under the terms of the Creative Commons Attribution License (https://creativecommons.org/licenses/by/4.0/).

insight from this study comes from establishing that the signaling dynamics in the CE are not in steady state and proposing that the movement of the source may have to do with this lack of stationarity. Perhaps I have missed something but I think this is an important point to clarify conceptually.

R1. We believe the reviewer has not missed the conceptual novelty of our paper! (she/he states the message concisely as “the signaling dynamics in the CE are not in steady state and proposing that the movement of the source may have to do with this lack of stationarity.”). We describe the signaling signatures of organs in which the Hh source is either (spatially) static or mobile and explain the differences as arising “ from the same signaling network but differ qualitatively due to the source movement”. We further speculate that multiple uses of the Hh cascade in different organs might be related to the versatility in signaling outputs it allows.

2- A second major concern is the way the model is employed in this study. I list my concerns below:
2.a. The model assumes 14 parameters (listed on Table 1) and the values these parameters take are important as they determine how fast the signaling dynamics are relative to the source movement. I found the arguments the authors use for the parameters they chose not convincing.
a. The diffusion coefficient of Hh D should follow D = \lambda^2 * k where \lambda is the decay length of Hh is and k = (1/60)mins (13um according to a study the authors cite). Is this right? This does not give a D of 0.1um^2/min.
I would suggest writing down the calculations the authors have performed to get the diffusion coefficient to avoid confusion.

R2.a. Sorry for this confusion. The value of D is not calculated, it comes from experimental measurements (Garcia-Morales et al., 2019). To calculate the value of D using the value of lambda from our previous publication (Miguez et al Current Topics in Developmental Biology, Volume 137, 2020, Pages 307-332), we would also need an accurate estimation of k, as the referee correctly suggests. In this case, k does not only account for the natural degradation of Hh, but also the rate of disappearance due to the intake by the cells. Since an *in vivo* quantification of the value of D is available, we decided to directly use this value, instead of indirect estimation via lambda and k. We have included the reference to (Garcia-Morales et al., 2019) when describing D in the manuscript to make sure the source of this value is identifiable.

b. How the authors have estimated binding and unbinding rates of Hh to its receptor is also not clear. They make a brief mention of affinities but they don’t explain how the rates were inferred from the affinities. Can you please clarify?

R2.b. Previous data from our lab have shown that, in presence of high levels of Hh, almost all (around 90%) of the Ptc in a cell is bound to Hh, therefore there is a high affinity of Hh to Ptc (Miguez et al., 2020). The way we incorporate this observation into our model is by calibrating the values of k1 and k2, in such a way that most of Ptc is bound to Hh during the process. This is achieved by tuning the affinity and dissociation rates of both molecules, to ensure that, as soon as Hh is available, a strong link (high affinity and low dissociation) between Hh and Ptc is established. In the present version of the manuscript we have added a sentence to clarify how these two values are estimated.

c. Importantly, I do not see how the degradation rates for Hh, Ptc and the Hh- Ptc complex were estimated. The table simply states “calibrated”. What does this mean? These parameters are very important as they determine whether the timescale in which the signaling dynamics reach a steady state. I suspect there is no obvious way to obtain these values directly in which case I think a better use of the model is to perform a phase-space analysis to explore the parameter regions in which the authors’ conclusions about the significance of the Hh source movement are valid.

R2.c. This is a very good observation. As the referee mentions, it is really difficult to estimate these degradation rates experimentally. Some values can be obtained *in vitro*, but they can

© 2022. Published by The Company of Biologists under the terms of the Creative Commons Attribution License (https://creativecommons.org/licenses/by/4.0/).

differ orders of magnitude when proteins are *in vivo*. Therefore, our approach is to select a range of parameter values and to compare the dynamics of the model with the dynamics observed in the experiments. This way, the values of some parameters are adjusted, in order to match the dynamics observed in the experiment (this is what we mean by “calibration”).

In the particular case of degradation rates, we assumed a slow degradation rate relative to the 300 min period considered. For the submitted manuscript we verified that changes in one order of magnitude in these degradation rates do not substantially affect the results.

However, and motivated by this suggestion, we have performed an exploration of the parameter space, where we vary several key parameters such as production rates (which is similar to varying degradation rates, since the balance between the two processes sets the protein levels), above and below a 50% of the value used, and explore whether the peak separation also takes place. This analysis is now shown as part of a new supplementary figure 4, and indicates that the effect of separation of peaks is robust, as it occurs across a wide range of parameter values. Interestingly, some of the changes result in the opposite effect expected (for instance, increase in Smo levels result in decrease in CiA levels), probably due to the effect in the dynamics of the feedback. In the same direction, and motivated by another of your suggestions (see below) we have seen that the peak separation is inhibited for a very wide range of values for the boundary velocity, and that it only occurs if the boundary is very slow or static.

© 2022. Published by The Company of Biologists under the terms of the Creative Commons Attribution License (https://creativecommons.org/licenses/by/4.0/).

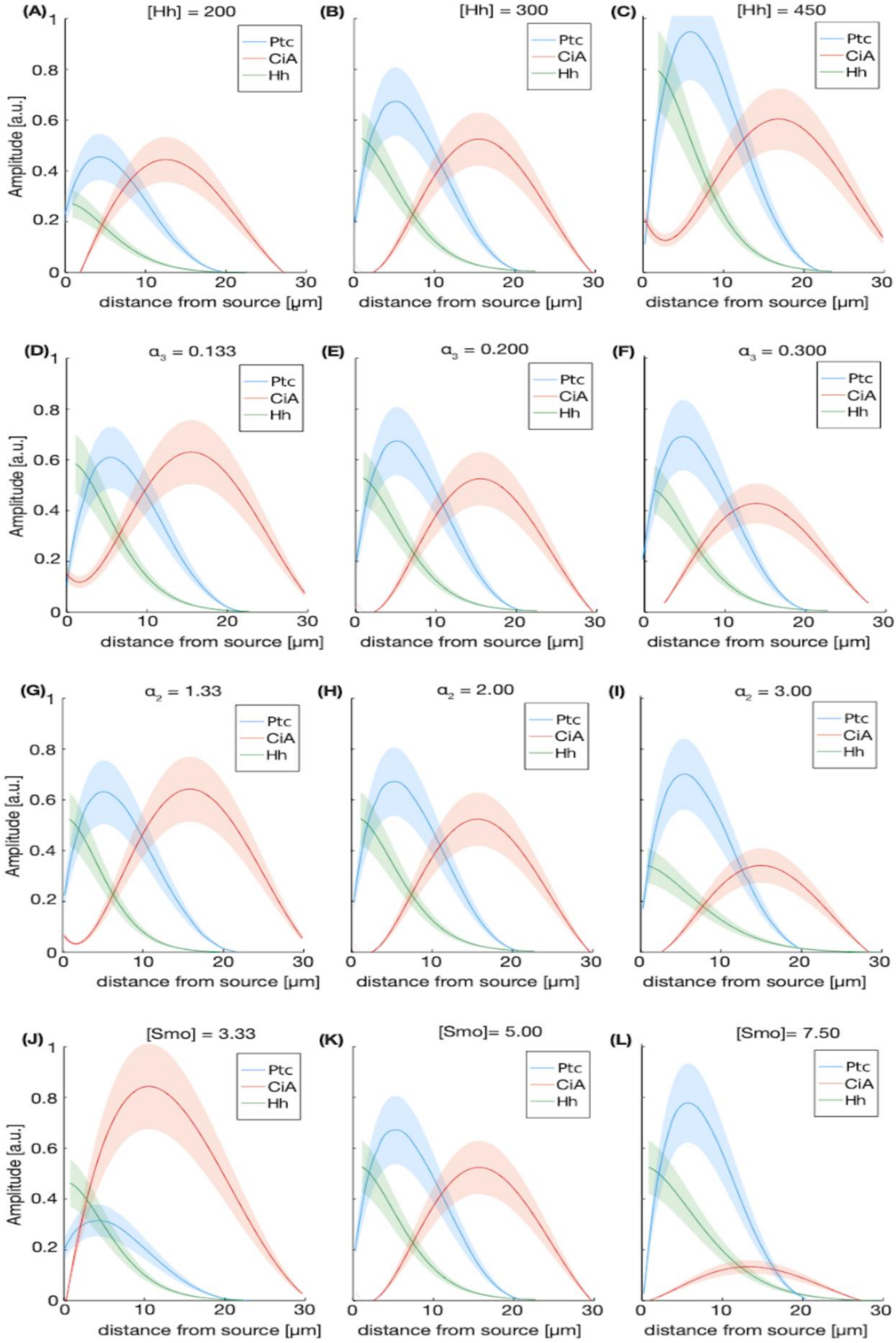

© 2022. Published by The Company of Biologists under the terms of the Creative Commons Attribution License (https://creativecommons.org/licenses/by/4.0/).

Caption Supplementary Figure 4: (A-C) Profiles of Hh (green), Ptc (blue) and CiA (red) for boundary levels of Hh 50% below (A) and above (C) the value used in our simulations. (D-F) Profiles for production rates of Ptc 50% below (D) and above (F) the value used in our simulations. (G-I) Profiles for activation of CiA 50% below (G) and above (I) the value used in our simulations. (J-L) Profiles for levels of Smo 50% below (J) and above (L) the value used in our simulations. This analysis shows that the feature of peak separation in the static signature profile is robust against changes in the key parameters of the model

d. The Hill coefficient for the feedback function in the signaling dynamics was also “calibrated”. What does this mean? Again, this is a parameter that is likely to impact the overall dynamics and steady state as it affects the sharpness of the feedback.

R2.d. The referee is correct in that the value of the Hill coefficient can be very important, because it sets the slope of the transition between no activation to activation (or no repression to repression). In our case, the overall final expression profile is quite robust to changes in the value of the Hill coefficient, and apart from situations of no cooperativity (m=1) or extremely sharp and unrealistic transitions (m>50), the shape and relation between Hh, Ptc and CiA profiles is maintained. Therefore, for the final simulations here shown, we have chosen an intermediate value of m=5.

Another, and perhaps more important parameter, is the value where the effect of the Hill function is half maximal (M2 and M3). The final values chosen for these parameters are estimated by performing numerical simulations and monitoring the max and min levels of the substrate molecule in each case. Then, M2 and M3 are set as the middle point between these max and min values. This way, we calibrate the transition between no activation and activation (or no repression to repression) to occur inside the range of values of the substrate molecule.

Sorry for not being clear enough about this process in the previous version of the paper. We have updated the Methods section with a more detailed explanation of how the parameters are selected.

e. How were the Hill coefficients of Ci and Ptc expression estimated? What are the confidences for these values?

R2.e. This point is related to the previous one. In the new version of the text, we explain how these and other values are chosen. For these two particular values, we have performed simulations to estimate the range of values that still provide a similar peaks separation signature, characteristic of the static situation (wing, ocelli and antenna). For the value of the Hill coefficient, any value between 2.5 and 20 results in a clear peak separation (the value used in our simulations is 5). For M2 (half maximal activation of expression of CiA mediated by Smo) the range is from 3.3 to 7.5 (around 50% up and down the value used of 5). For M3 (half maximal repression of the expression of Ptc mediated by CiA), the range is 1.6 to 50 (4 times smaller and 10 times larger than the value of 5 used).

f. I can see how some parameters may not be easily quantified and this is ok. I think what is important is that authors clarify how important their choice of parameters is for their conclusions (given that many parameters in the model are free) and explain clearly how parameter values were estimated, calibrated or inferred from the literature.
R2.f. We fully agree with the referee on this point. Some parameters are difficult to quantify. Also, in these types of simplified models, a given parameter may condensate several interactions. In these cases, a good option is to tune the parameters until the dynamics predicted by the model resembles the one measured in the experimental condition, and then use the model to predict changes and variations from this condition.

Following your suggestion, we have updated the Methods section with a more detailed explanation of how parameters have been chosen.

g. It would be useful to see a comparison of the model and the experiments when the model considers a moving source. Fig. 4 shows a qualitative picture but it would be much more informative if the authors plotted the signaling and ligand dynamics in this case and then

© 2022. Published by The Company of Biologists under the terms of the Creative Commons Attribution License (https://creativecommons.org/licenses/by/4.0/).

repeat the simulations with a progressively slower moving source. Would this lead to the peaks becoming progressively more separated? It would be good to also recapitulate the mutant/RNAi versus control dynamics and quantifications in Fig. 4 with the model using a static, fast and slow moving source with all other parameters kept the same.
R2.g. Following this suggestion (also suggested by the other referees), we have added a new supplementary figure (Suppl. Fig. 2) with the profiles at different time points.

Focusing on the analysis suggested by the referee to test different speeds of the furrow, a result of this analysis is also part of a new supplementary figure. In brief, we see that peak separation occurs only below speeds of the furrow of 0.04 cell/ hours (i.e., 20 times slower than the experimental estimation of 1 cell diameter/hour). At around 0.04 cells/ hour we recover the intermediate situation shown in the mutant eye conditions, while above this value, there is no peak separation. Now we have estimated roughly the deceleration of the differentiation wave induced by attenuating *ato* (in CE discs of “mutant” ey>ato_RNAi larvae, as the proportion of ommatidia differentiated until late larva of mutant CEs relative to controls) to be 1/3 of the control speed. The discrepancy between the computational 1/20 and the experimental ⅓ is likely the result of specific parameters choice but agree in that the signaling profile is quite sensitive to the movement of the signaling source. (See below, were we reiterate this point).

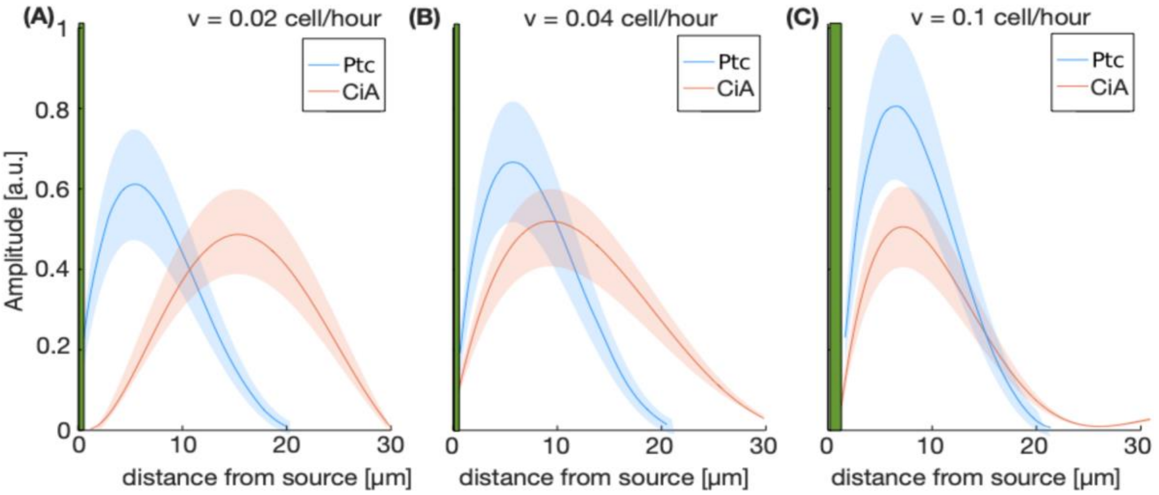

Supplementary Figure 3: Profiles of total Ptc (blue) and CiA (red) levels at t =300 min for different velocities of the Hh boundary (moving from left to right at constant speed). This analysis shows that, for the parameter values used, only speeds below 0.04 cell diameters/hour result in a clear peak separation, typical of the signature profile of antenna, ocellus and wing (static Hh sources). Velocity values around 0.04 cell diameters/hour (panel B) show an intermediate situation similar to our experimental results of mutant eye condition (*ey>ato-RNAi;* Figure 4A in main text, central panel). Values above this threshold result in a distribution of peaks that resembles the CE signature profile (moving source).

h. This is a more minor point but I find the notation used for constructing the model confusing. Instead of using the molecular names as parameters I suggest defining simple letters (e.g. C for concentration, R for receptor, S for signal or something along these lines). It’s currently quite hard to read the equations and figure out which letters are indices and which are part of a parameter or variable. Also since only one diffusion coefficient is used the subscript is not necessary for D_1.

R2.h. We apologize for the complexity in the understanding of the equations. We have tried the suggestion of the referee and we feel that the resulting equations are not much more clear (we have one receptor R (Ptc), one ligand L (Hh), and two signals (CiA and Smo)). We feel that the nomenclature suggested by the referee obscures somehow the formation of complexes (HhPtc will be now LR, and SmoPtc will be now SR). Also we feel that keeping the names for the variables similar to the names of the proteins they represent may facilitate the analysis for experimentalists familiar with the Hh pathway. Finally, this solution allows a more direct link between the interactions in Fig 3 and the equations.

© 2022. Published by The Company of Biologists under the terms of the Creative Commons Attribution License (https://creativecommons.org/licenses/by/4.0/).

On the other hand, we tried to simplify as much as possible the equations (removing subindexes when not necessary). We also indicated in the text that the first equation is solved globally, while the rest of the equations are solved independently in each cell. Finally, we are using for displaying the equations a more modern typography that we think improves readability. We hope that this new version of the equations is clearer.

3. Relevant to some of the concerns I raised regarding the model and given that the assumption that the source in the CE moves faster than the signaling dynamics, is there an experiment that could provide an estimate for the time scale of the signaling dynamics downstream of Hh? FRAP or pulse-chase come to mind, are these or other experiments that tackle this point feasible?
R3. This point was also raised by reviewer#1, and indeed, it is a very interesting point, but one about which we can only speculate. Although some biophysical or biochemical experiments could be designed, perhaps the best estimates of global rates can be extracted from the dynamics of biological processes. At 25ºC, the differentiation wave advances roughly one cell diameter per hour (that is, everything necessary for the induction of R cells and their production of signals, including Hh, happens at that pace). In our dynamical system, the signal needs to first activate CiA (biochemical cascade), this is then followed by an increase in Ptc (transcriptional/translational process) and then finally a downregulation of CiA (biochemical cascade) occurs. At least transcription and translation happens on the scale of minutes. And even if generally biochemical events might happen much faster (seconds), it is possible that some of the biochemical processes involved in Hh signal transduction require subcellular relocalizations, which might be more sluggish. So, very roughly, it might be that the timing imposed by the pace of retina differentiation (1 hour) is, indeed, tight for the loop to be closed. In some additional experiments carried out for the revision of this paper, we have induced the expression of Hh ectopically in the wing pouch. Although the time resolution of the process afforded by this experiment is not great, the fact that we find discs in which the ectopic Hh expression is very clear and yet the separation of the CiA and Ptc peaks has not occurred (something that does happen in this experiments in later time points) suggests to us that the "peak displacement" of Ptc and CiA maxima is not fast (see new Figure 4). Therefore (at 25ºC), transcriptional responses to Hh signaling occur within 1 hour, while the adjustments of CiA and Ptc peaks need longer times.

Another issue is that the Ptc and Ci peaks in the RNAi experiment do not become separated in the experiment as predicted by the model. In fact, the authors only compare the amplitude of Ptc and the decay of Ptc in the two conditions. I guess this could in principle be explained if the dynamics is still not at steady state with a slow- moving source. Can the authors elaborate on this point? Can the speed of the source in the RNAi conditions be quantified and compared to the control?

This point has been answered thanks to the above suggestion of this referee. The model has shown us that only a full stop will result in a complete separation of the Ptc and CiA peaks, and perhaps this is the reason why the RNAi experiment only results in an intermediate solution. These observations have been added to the Discussion part of the new version of the manuscript. Regarding the second question: the retardation of the MF velocity can be calculated as the percentage of ommatidial rows assembled in mutant CE discs (ey>ato_RNAi) relative to those in controls, assuming a constant speed in either group. The number of ommatidia in late L3 CE discs is 17 ± 2 and 6± 3 in controls and mutants (N=10 in both groups), respectively. Therefore, the MF speed is reduced, on average, to 35% of the control. This is translated to about 1 cell diameter every 3 hours at 25ºC. We have included this estimate in the revised version of the manuscript.

Some minor points:

- Why were polynomial fits performed to the experimental profiles? Why not show the average of the 5 experimental profiles instead? If the authors insist to perform the fits then more details of the functions fitted, quality of fits and error estimations should be provided in the methods.

© 2022. Published by The Company of Biologists under the terms of the Creative Commons Attribution License (https://creativecommons.org/licenses/by/4.0/).

As the referee suggests, there are several ways to perform the average of spatial profiles, and when done correctly, all should provide very similar results (we are presenting a comparison between direct average between several profiles and average after polynomial fitting in a new supplementary Figure). We try several options, and we finally decided to use the average between polynomial fittings for the following reasons:

- Each profile for each individual experiment has been quantified in a region of slightly different size in the x-axis (see profiles in panel A of the new supplementary figure). To directly average between profiles, as the referee suggests, we will need to fill the shortest ones with zeros (or with a minimum value), to be able to sum vectors of the same length. If we directly average over the parameters of the polynomial fitting, we do not need to have all profiles of the exact same length.
- Since we are using here the average profiles mainly as a guide to the eye (so a reader can directly see the type of curve we are obtaining), we felt is was more informative in this respect a smooth curve (such as the one derived from the polynomial fitting) than a noisier type of curve that comes from the direct average of the profiles (panel B in the new supplementary figure).
- For consistency with the model. Since cells in the model are aligned in space, an average over several profiles will result in a sequence of 10 points (10 arrows of cells). Polynomial fitting results in a more continuous profile, which we felt was more informative, and for the sake of consistency, we decided to use the same approach when representing the experimental data.

Detailed information about how the fitting is performed, the type of fitting used, is not part of the Methods, as suggested by the referee. This information is also complemented by the new supplementary figure that allows us to illustrate how we obtain the profile.

Regarding the quality of fitting, we are including as supplementary material of the present version, a compressed zip file with all the residuals for each fitting performed in the experiments and in the model profiles.

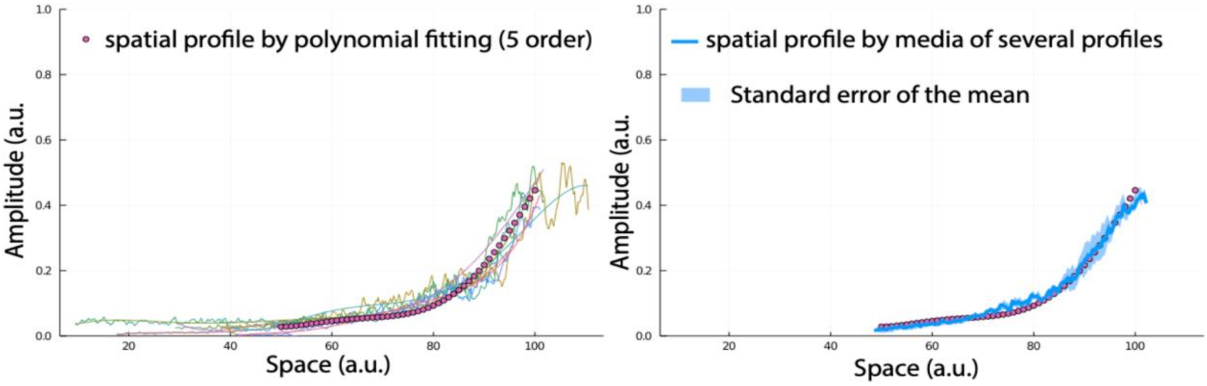

Caption Supplementary Figure 1: Example of calculation of average profiles from several experimental measurements. (Left panel) Several profiles are plotted for Hh, together with their corresponding polynomial fitting. Dotted line corresponds to a polynomial plot with parameters calculated from the mean value of all polynomial fittings of the individual curves. (Right panel) Comparison between polynomial average (dots) and the direct average (blue line) over 5 curves, showing a good agreement. Blue ribbon corresponds to the standard error of the mean, showing a small deviation that under-represents the variability in the profiles (this is why a 50% confidence interval is used instead).

- I wonder if it makes more sense to normalize the curves of the dynamics to make them more directly comparable. Are the differences in amplitudes in the experiments meaningful if different FPs are used?

Again, we thank the referee for pointing out this issue. We also debated several times whether to present the profiles normalized or not normalized. We finally decided to opt to not

© 2022. Published by The Company of Biologists under the terms of the Creative Commons Attribution License (https://creativecommons.org/licenses/by/4.0/).

normalize them since images are taken with the same microscope settings (laser intensity, gain…) so there was information about changes in intensity that we felt would be lost if we normalized the profiles.

• Can the authors report the p-values for all their statistical tests in the figure legend or methods?

The p-values are now reported as part of the figures.

• The ribbons in the experiments show the sem but in the model they show the 50% CI. Can the authors also show the sem in the model to make the plots comparable?

We apologize for this confusion. We tested several different ways to show the variability of the profiles, and finally decided for the 50% CI, (we felt that the SEM underestimated the real variability that we were seeing in the profiles). During the process of editing, we forgot to change the caption of Fig 1. We have corrected this typo in the present version of the manuscript.

• Fig. 4E is not easy to interpret. I would show the quantifications instead.

Following both suggestions from referees 1 and 2, now we also show the quantification as profiles as part of a new supplementary figure.

• Fig. 4C, shouldn't the exponent be dimensionless (not in a.u.)?

The referee is correct. We have corrected the legend in the now Fig 5C.

Reviewer 3 Advance Summary and Potential Significance to Field:
Hedgehog (Hh) acts as a patterning morphogen in a variety of developmental contexts. A universal feature of Hh signalling is the upregulation of its receptor Ptc and this feedback self-limits its range of action. In this manuscript, the authors investigate the patterns of Hh-GFP, Ptc, and the signalling effector CiA in the Drosophila compound eye (CE), wing, ocelli, and antenna. They found that the spatial peaks of Ptc and CiA differ in the CE with respect to the other tissues examined. Using a mathematical model, the authors observe that an early time point in the dynamics of the signal somehow resembles the patterns in the CE and suggest that this is due to the moving source of Hh, driven by the progression of the morphogenetic furrow (which does not occur in the other organs). The authors then attempt to test this hypothesis experimentally by inhibiting genetically the progression of the morphogenetic furrow.

Reviewer 3 Comments for the Author:
In my opinion, the mathematical analysis is not very useful and the conclusions of the manuscript are not necessarily supported by the evidence that is presented (see below). Therefore, I do not support publication of the manuscript in its current form.

1. In Fig. 2 the authors quantify differences in the profiles of Hh-GFP, Ptc, and CiA in the CE, Antenna, Ocellus, and Wing. They focus on the following two qualitative observations of their data: (i) "The signaling profiles of Ptc and CiA in the wing, antenna and ocellus were qualitatively similar, with a first peak of Ptc followed by a peak in CiA further from the Hh source. On the other hand, the signaling signature of Hh in the CE was qualitatively different from the others, with a peak expression of Ptc that is coincident in space with a maximum in CiA". (ii) "the signal intensity of Ptc at this maximum was significantly lower in the CE that in the other tissues."

The authors claim that these findings are counterintuitive and they justify the need to build a mathematical model to make sense of these data, but the reality is that there is little need of the mathematical model since it displays exactly what would be expected by intuition. Because Ptc is a repressor of the system, it is expected that a peak of Ptc near the Hh source will shift the expression of CiA.

© 2022. Published by The Company of Biologists under the terms of the Creative Commons Attribution License (https://creativecommons.org/licenses/by/4.0/).

The referee, similarly to referee#1, suggests that finding non-coincident Ptc and CiA peaks is not totally surprising in an spatially extended dynamical system with a negative feedback. We have to admit that this intuition was not obvious to us when we started this work, especially because this is a spatially extended system when thinking that the spatial response of the signaling components (Ptc and CiA) depends on a Hh gradient that is dynamically modified by the signal (through Ptc). In addition, as Ptc is a CiA target, decreasing CiA should be followed by a reduction in Ptc levels. Actually, the separation of the peaks is observed in our computational model only for a subset of parameters. In any case, though, we have posed the problem we study more clearly by modifying the sentence as "The observation of non-coincident peaks for CiA and Ptc in wing, antenna and ocellus might not have been surprising in a spatially extended system with Ptc exerting a negative feedback, but why is it not observed in the CE?".

In figure 2B of the CE, it is not clear if x=0 corresponds to the position of the morphogenetic furrow (source of Hh-GFP), or how is x=0 defined? From the profile of Hh-GFP it appears that there is a non-zero plateau of Hh expression at least for the first 10 microns. If these cells are producing Hh, then it is expected that Ptc levels are lower in this region since more Hh is binding free Ptc in the system.

We apologize for not being sufficiently clear in the explanation of the process of the quantification of the profiles. To align all different profiles, a reference point needs to be defined to compare the amplitude levels of Hh, Ptc and CiA across the different tissues and conditions. This location (defined as x=0) is defined as the point when levels of Hh start to decrease, as marking that we are outside of the region of Hh producing cells and into the region of Hh sensing cells. In this way, we are only comparing the features in the region of Hh sensing cells in all tissues (x>0).

We updated the manuscript to include this information, as part of the Methods section, explaining that the decrease from this plateau levels is used to define the value x=0, as explained.

2. The authors then solve their mathematical model using parameters from previous studies and observe that at earlier stages of the dynamics the peaks of Ptc and CiA coincide, but at later times these peaks start to separate from one another. This led the authors to propose "the model reproduces the profiles for the CE and the other organs as an early and a later time point, respectively, along its temporal dynamics". This is a major flaw of the paper. First, because the profiles of Fig. 2C (CE) and Fig. 3D do not look that similar as they claim. But the main problem here is that even if we accept that they look similar, there is no evidence that the CE corresponds to an earlier stage of Hh signalling dynamics. There are other plausible explanations of why the peak of CiA does not separate from the peak of Ptc expression in the CE (as I said in the previous point, this is most likely due to cells gradually becoming source of Hh, which reduces the levels of free Ptc although these do not become sufficiently strong to repress the expression of CiA). But there are other explanations that could be considered if they want to use their model as an exploratory tool. For example, from Fig. 2, it appears that the levels of Hh are different in each of the organs; this will also affect the levels of Ptc that will be expressed in receiving cells; having different Hh levels at the source could affect the dynamics without affecting the network.

To explore if changes in Hh would have an effect on the signaling profile, and potentially could explain the differences between Compound eye and the other tissues, we have performed numerical simulations where the values of Hh at the source have been modified. Results are shown in a new supplementary Figure (Supp Fig 4), where Hh levels have been modified 50% below (Supp Fig 4A) or above (Supp Fig 4C) the value used for our simulations (Supp Fig 4B, reproduced below). Comparison between these plots show that the separation of peaks does not depend substantially on the levels of HH.

We also studied the same dependence when the production of Ptc is changed below below (Supp Fig 4D) and above (Supp Fig F) the value used for our simulations (Supp Fig 4E). These results also show that the peak separation is very robust against changes in the levels of Ptc production that also affect the final levels of CiA, as expected.

© 2022. Published by The Company of Biologists under the terms of the Creative Commons Attribution License (https://creativecommons.org/licenses/by/4.0/).

To complete this study of robustness, inspired by this referee suggestion (and also by a suggestion of referee 2 to perform a phase space analysis), we also decreased (Supp Fig 4G) and increased (Supp Fig 4H) the production of CiA by 50%. Again, the results show that the separation of peaks does not depend strongly on the levels of CiA (surprisingly, increasing production of CiA results in final lower intensity levels of CiA, perhaps due to the negative feedback). Also, dependence on Smo leves is also studied (Supp Fig 4J-L), where we can see that despite the large changes in levels of CiA, the PtC and CiA peaks also separate clearly. Again, the dependence is opposite as expected, (CiA levels reduce when we increase Smo), again due to the effect in the dynamics of the feedback by Smo. All these results are presented in a new Supplementary Figure 4 (see below).

In addition, we have carried out an experiment that addresses directly the time- evolution of the Ptc and CiA profiles (now as a new Figure 4). In it, we follow these profiles in wing discs where a tagged version of Hh is expressed. While in early discs the two peaks coincide and straddle the Hh-expressing cells, in later ones Ptc remains centered on the Hh-expressing cells while the CiA maxima displace. Therefore, at least in this ectopic expression assay, the expression profiles that our model generated as a temporal succession, are also a temporal succession *in vivo*. Now the text includes the following description of results:
"To test this computational prediction, we expressed a GFP-tagged form of Hh (Hh- GFP) ectopically in the anterior region of the wing disc (where Hh is not produced, but received) and followed the response of the Ptc and CiA signals. As driver, we used the c96-GAL4 line, whose expression initiates in mid-third larval stage (mid-L3; see Material and Methods) in a domain that crosses the wing pouch (Figure 3). In mid-L3 discs, around the time of Hh-GFP expression initiation, the Ptc and CiA signal increases straddling the Hh-GFP cells. However, in late L3 discs, while the Ptc peak remains centered on the Hh-GFP domain, the CiA maxima are displaced (Figure 4).
Therefore, this ectopic-expression assay reproduces the temporal dynamics of the Ptc and CiA profiles predicted by the model."
The new results are included below. The Materials and Methods section has been expanded to include a full description of the experiment.

© 2022. Published by The Company of Biologists under the terms of the Creative Commons Attribution License (https://creativecommons.org/licenses/by/4.0/).

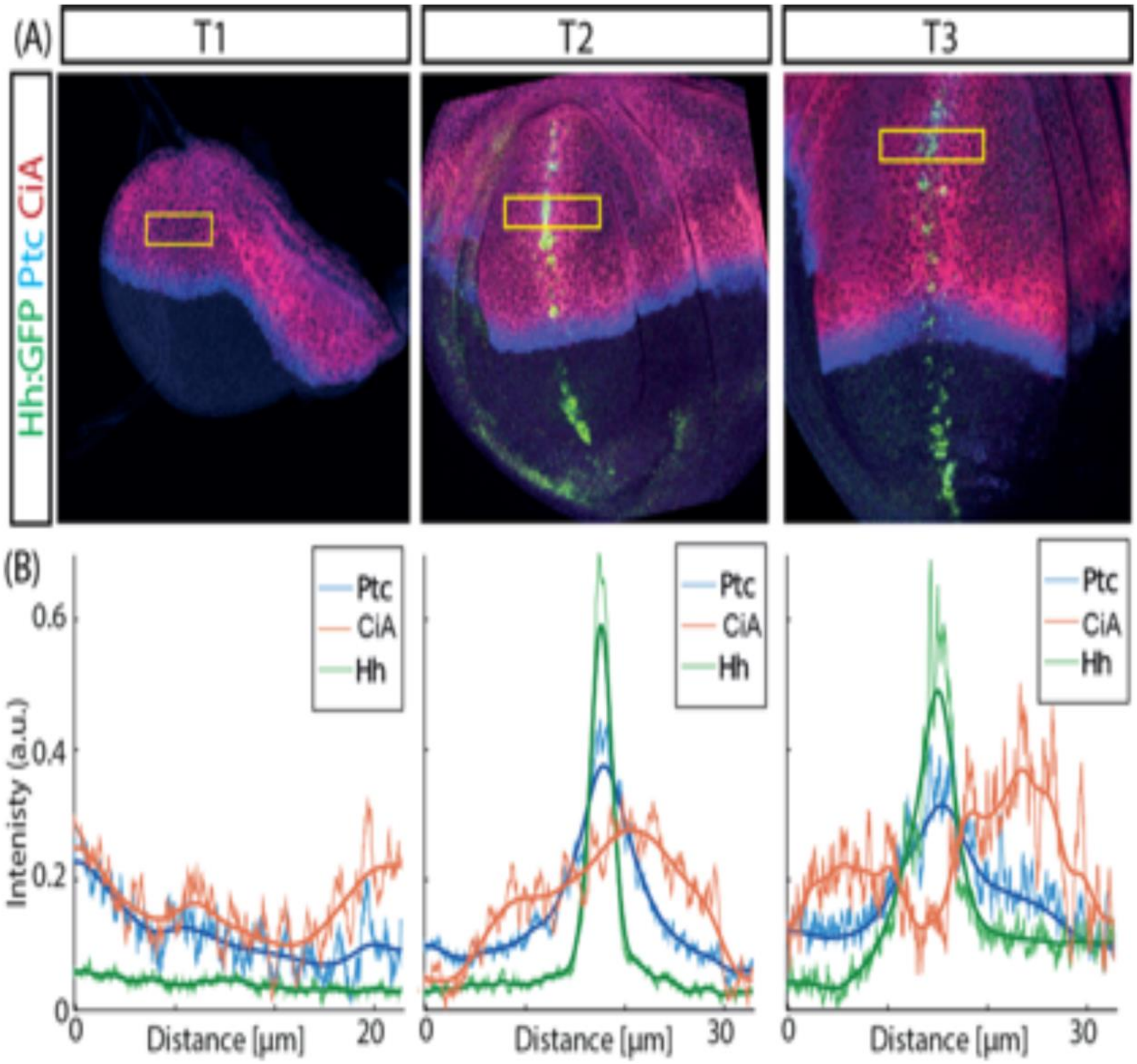

Progressive separation of Ptc and CiA signal maxima in wing discs with ectopic Hh expression. (A) Expression profiles for Ptc and CiA were obtained from similar regions of the anterior compartment of the wing disc pouch (boxed) from progressively older discs (T1-3). Expression of Hh-GFP driven by c96-GAL4 along the DV wing disc boundary initiates in mid-L3 discs (T2). At earlier times there is no Hh:GFP expression (T1). (B) Ptc, CiA and Hh:GFP expression profiles from the boxed region in early (T1), mid (T2) and late (T3) representative wing discs centered around the c96-GAL4 expression domain. The actual profiles and the denoised curves (thicker lines) are shown. After initiation of induction, there is an increase in CiA and Ptc expression with coincident maxima straddling the Hh:GFP domain (T2). In the T3 disc the CiA maxima are displaced laterally relative to the Ptc peak (T3).

© 2022. Published by The Company of Biologists under the terms of the Creative Commons Attribution License (https://creativecommons.org/licenses/by/4.0/).

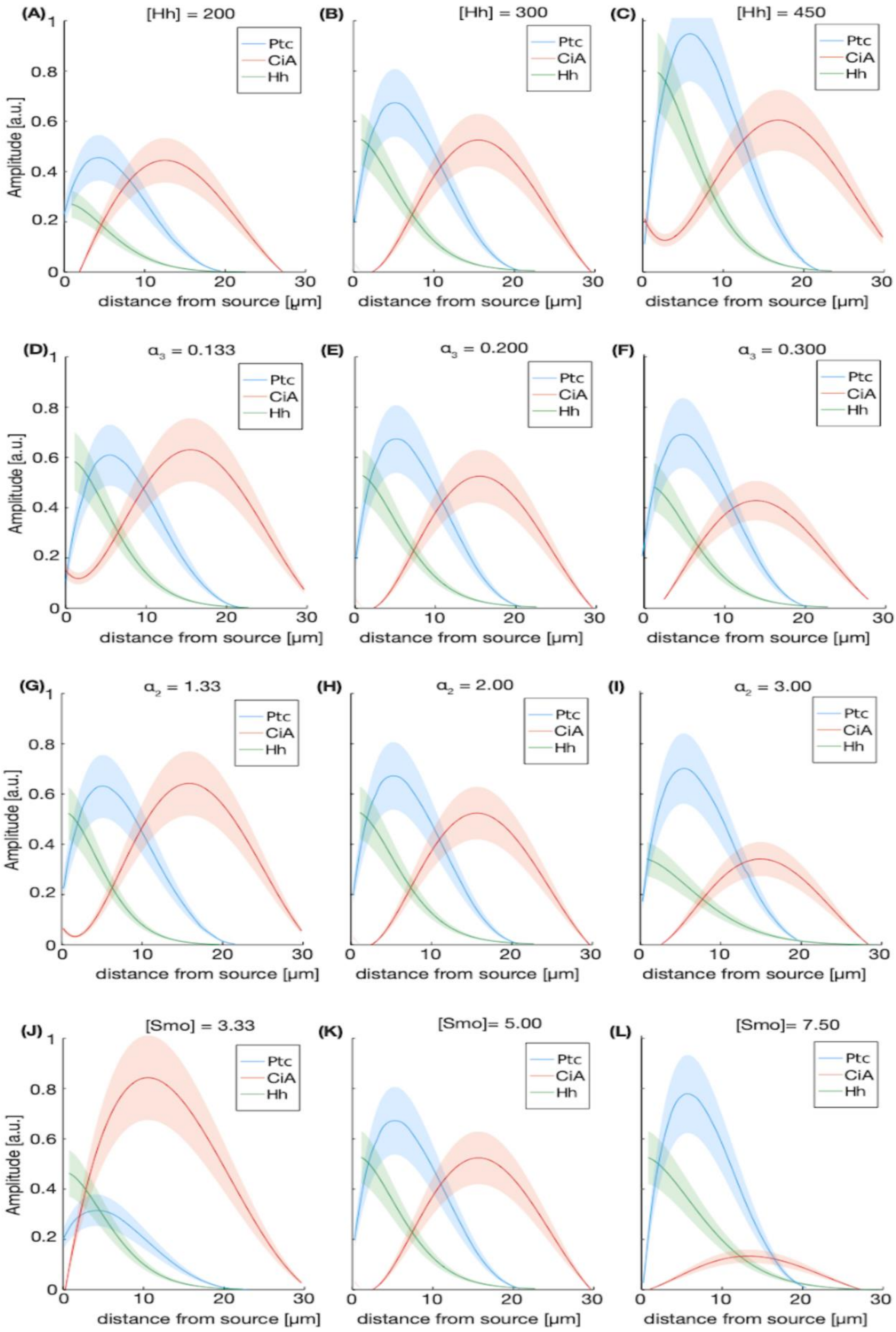

© 2022. Published by The Company of Biologists under the terms of the Creative Commons Attribution License (https://creativecommons.org/licenses/by/4.0/).

Caption Supplementary Figure 4: (A-C) Profiles of Hh (green), Ptc (blue) and CiA (red) for boundary levels of Hh 50% below (A) and above (C) the value used in our simulations. (D-F) Profiles for production rates of Ptc 50% below (D) and above (F) the value used in our simulations. (G-I) Profiles for activation of CiA 50% below (G) and above (I) the value used in our simulations. (J-L) Profiles for levels of Smo 50% below (J) and above (L) the value used in our simulations. This analysis shows that the feature of peak separation in the static signature profile is robust against changes in the key parameters of the model.

3. The experiment in Fig. 4 in which the Gal4-UAS system is used to reduce the movement of the morphogenetic furrow shows that in fact the levels of Ptc in receiving cells and the differences in the peaks of Ptc and CiA are related to the movement of the source of Hh, but this may have nothing to do with the dynamic state of the signal (which is the main conclusion of the paper). Once again it may have to do with the levels of free Ptc that ramp up when receiving cells are not allowed to become source cells. Therefore, I do not find that this experiment supports the authors' claims.

In fact, we believe that the interpretation we make of our results is *the same* as the referee's! We are analyzing the Hh-signaling profile of *Hh-receiving* cells. In case the source does not move, signaling progresses temporally from overlapping peaks to separated peaks in time (something predicted by our model, now verified experimentally, (see new figure 4 of the manuscript). If the source moves, receiving cells do not spend *as "receiving"* enough time before becoming "producing" (i.e. Hh source). In the CE, the time a cell spends as "receiving" depends, all other things being equal, on the time it takes for it to become "producing". This time comprises the period since first reception of Hh signaling (and activation of *ato*) until the cell becomes Hh- producing itself. If the pace of this process is fast enough, there will not be sufficient time for the signaling to complete the feedback loop and reach a quasi-steady state characterized by the separation of Ptc and CiA maxima. Now we include an estimate of the relative reduction in differentiation velocity in *ey>atoRNAi* (mutant) CE discs: the velocity in mutants is about one third that of controls. At this lowered pace, the loop would have time to operate, since we observe a tendency towards an increase in Ptc and the displacement of the CiA max. Therefore, it seems to us that both the referee and ourselves are giving equivalent explanations.

In addition, this experiment was not done rigorously and the data are not presented adequately. They use different temperatures to control the strength of the Gal4 system, but they should use the Gal80ts to provide a more robust time control of atoRNAi expression. Actually the use of the Gal80ts may be used to assay the patterns of CiA and Ptc in early vs. late signaling.
We followed the referee's advise and designed a number of experiments to test the potential origin of the distinct signaling patterns in temporal differences. After many attempts (many of our stocks had been lost during the covid period, many of those stocks, when received from other colleagues wouldn' work as expected (likely because the stocks had also got contaminated)) we found an experiment with which to test this hypothesis -the one described above-, which allowed for spatially clean expression and a temporal progression. We believe that this experiment now provides solid evidence for the hypothesis that the two signaling profiles are but the temporal evolution of the same dynamics (new data on figure 4 of the manuscript, reproduced below).

© 2022. Published by The Company of Biologists under the terms of the Creative Commons Attribution License (https://creativecommons.org/licenses/by/4.0/).

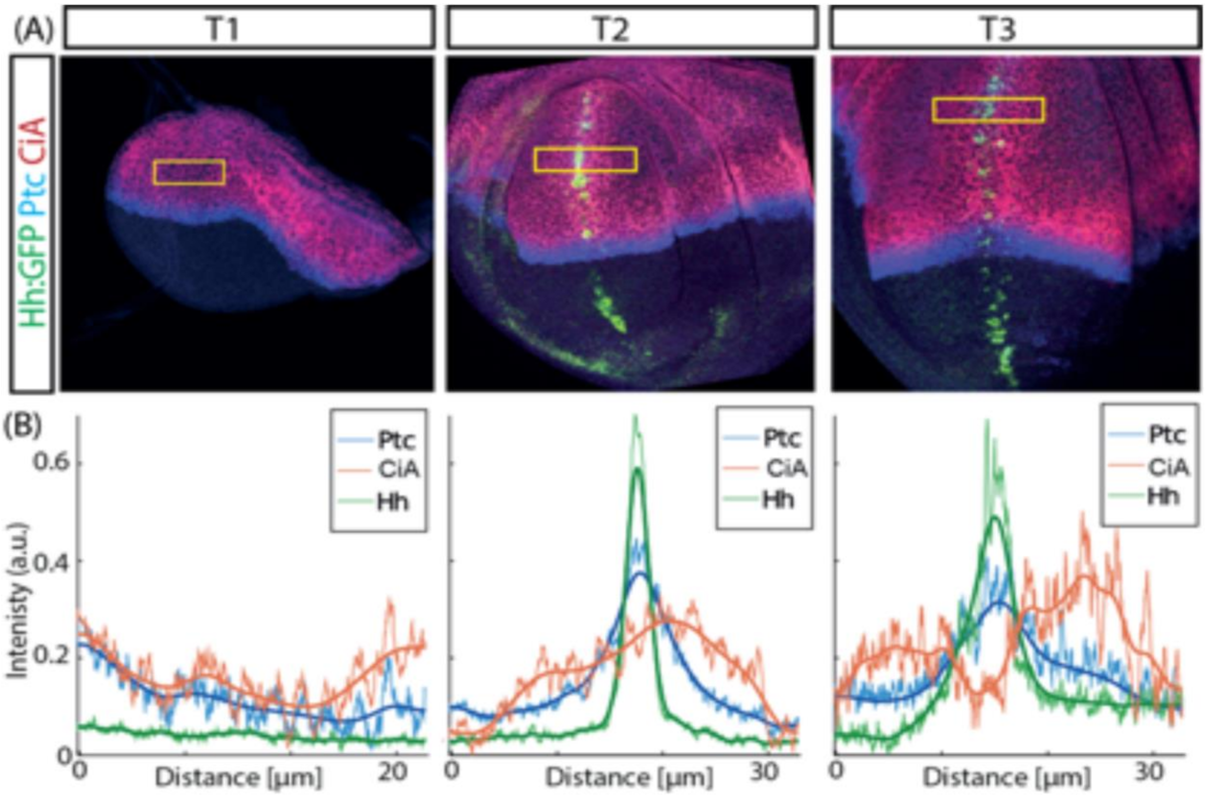

Progressive separation of Ptc and CiA signal maxima in wing discs with ectopic Hh expression. (A) Expression profiles for Ptc and CiA were obtained from similar regions of the anterior compartment of the wing disc pouch (boxed) from progressively older discs (T1-3). Expression of Hh-GFP driven by c96-GAL4 along the DV wing disc boundary initiates in mid-L3 discs (T2). At earlier times there is no Hh:GFP expression (T1). (B) Ptc, CiA and Hh:GFP expression profiles from the boxed region in early (T1), mid (T2) and late (T3) representative wing discs centered around the c96-GAL4 expression domain. The actual profiles and the denoised curves (thicker lines) are shown. After initiation of induction, there is an increase in CiA and Ptc expression with coincident maxima straddling the Hh:GFP domain (T2). In the T3 disc the CiA maxima are displaced laterally relative to the Ptc peak (T3).

Moreover, the authors do not show any image of the actual data (stained discs) that supports the results of panels B and C. In fact, panels B and C are unnecessary because the information can be easily obtained from A.
We considered that it was more informative to the reader to provide the average profile over several independent repeats of the experiments, (so we can also illustrate the average and the confidence interval). Also, we sincerely believe that the quantification of maximum Ptc and exponent allows us to support our analysis that differences between antenna and control are significant (p-value < 0.01) but non-significant when compared to mutant eye (p-value > 0.01).

In summary, the differences in the spatial distributions of the peaks of Ptc and CiA in the CE with respect to the other tissues are not necessarily related to an earlier dynamic state of the signalling.
After revision and further discussion, especially after including the new experiment in Figure 4, we hope that the referee now agrees with us that, at least, the interpretation that we offer (and which we believe coincides with her/his reading) is plausible and furthermore, parsimonious.

4. In the discussion, the authors state that their data supports that in the wing/antenna/ocellus, Hh signalling works as a traditional morphogen, whereas in the CE it does not. However, by looking at the profiles it seems to me that this is quite the opposite. In fact, it is in the CE that the CiA profile (which is the transcriptional effector of the signal) looks more graded (see

© 2022. Published by The Company of Biologists under the terms of the Creative Commons Attribution License (https://creativecommons.org/licenses/by/4.0/).

Fig. 2B). Actually, the fact that there is a stronger peak of Ptc in the wing/antenna/ocellus is more against the traditional morphogen hypothesis than in the CE. (In fact, a previous study in the wing by Nahmad and Stathopoulos (2009) that the authors cite and other papers by Briscoe and colleagues in the vertebrate neural tube do not support that Hh is a traditional morphogen).

A morphogen, operationally, is a molecule that dictates different cellular outcomes depending of the concentration received by these responding cells. While in the wing, at least, it is clear that Hh works as a morphogen (activating different targets at different distances from its source), in the CE, Hh regulates only one fate, at least anterior to the MF. Therefore, Hh "works" as a binary switch, rather as a morphogen, in the CE, despite the fact that its distribution and the expression of its signaling targets were graded or not. Mechanistically, the idea of a static morphogen gradient, as propounded by Wolpert, was surpassed years ago, when feedback loops were discovered. This fact added time to space, making signaling something not only space- but also time-dependent. This is, for example, what Nahmad and Stathopoulos discovered -that the Hh gradient shows a spatial *and* temporal dynamics that results in a transient signal "overshoot". Whether this overshoot carries additional information to the later quasi-steady state in the wing disc is not clear. Perhaps the time during which the cells affected by the overshoot are exposed to Hh is insufficient to drive changes in cellular behavior -or perhaps not. The experiments by the Briscoe lab do not go against neither the effective nor the mechanistic morphogen concept. It adds to it the fact that biochemical processes have "memory" and that, rather than instantaneous concentrations, cells are likely computing the integrated signal over the exposed period. Therefore, there is a correlation between the signaling profiles we find and the roles played by Hh, either as a morphogen or as a binary switch. In any case, we generally agree with the referee that the "morphogen" concept and how morphogens act is a very nuanced one. As an example, see the new discoveries on Shh action on digit patterning (Zhu et al., Dev. Cell 2022: 10.1016/j.devcel.2022.07.016).

Other comments:

-In the introduction, some key references are not cited. For example, the idea that Hh-dependent ptc upregulation self-limits the range of the Hh gradient in Drosophila was originally proposed by Chen and Struhl, 1996.

Thank you for pointing this out. We shouldn't have missed that reference, that now has been introduced in the manuscript.

-The model uses a Hill function for the activation of CiA, even though this is not a transcriptional activation process. How does this choice affect the results?

The referee is correct. The fact that we are using a Hill function to represent the activation of CiA is a simplification of the true process of balance between CiA and its repressor form. As the referee surely knows, Hill functions are commonly used to model transcriptional activation, but they are also suited to model other biological processes [J. N. Weiss. The Hill equation revisited: uses and misuses. FASEB J., 11 (1997), 835841]. One of the most common is ligand-receptor binding, or oxygen to hemoglobin binding, which is actually the process used to derive the equation by Hill himself [A. V. Hill. J. Physiol, 40 (1910)].

Here, we use the Hill function in CIA not as an activation, but to model its equilibrium concentration between two states (CiR and CiA, where CiA+CiR = constant). This equilibrium situation depends on the amount of Smo, that shifts the balance between the two Ci forms). By simplifying this process using a Hill function, we can eliminate directly the variable CiR from the equations, reducing the number of equations and the number of parameters. This simplified approach was decided after comparing the simplified model with a previous version that incorporated a more realistic interaction scheme between CiR, CiA and Smo. If dynamics is considered fast enough (binding- unbinding, compared to a transcription process), the two time scales can be separated and the levels of CiA can be considered in equilibrium and, thus, we can use the Hill function as a useful simplification Smo-CiA interaction to the full model.

© 2022. Published by The Company of Biologists under the terms of the Creative Commons Attribution License (https://creativecommons.org/licenses/by/4.0/).

Also, the authors used the parameter values of M3=2.5 and M2=5. Therefore, it is not surprising that the peak of CiA extends beyond the one for Ptc as more signal is required to activate Ptc than CiA. What is the evidence of this choice in the parameters and how does the dynamics change in other parametric conditions?

The Hill coefficients were chosen by performing numerical simulations and monitoring the max and min levels of the substrate molecule in each case. Then, the Hill coefficient was set as the intermediate point between this max and min values. Sorry for not being clear enough about this process in the previous version of the paper.

Motivated by this suggestion (and also by that by Referee 1), we performed more numerical simulations to estimate the robustness of the results against changes in the values of M2, M3 and m. For M2 (increase in Ci mediated by Smo) the range is from 3.3 to 7.5. For M3 (production of Ptc mediated by Cia), the range is 1.6 to 50 (4 times smaller and 10 times larger than the value of 5 used). We also modulated the value of the Hill exponent, and any value between 2.5 and 20 results in a clear peak separation.

In conclusion, this analysis suggests that the conclusions are robust against changes in the parameters of the Hill functions. For simplicity, we have updated our simulations using the same value of 5 for the M2, M3 and m.

-In page 10, the authors say that “Figure 4B,C shows three snapshots of this scenario taken at three different time points”. Should say 4E.

Thank you. This has been corrected in the present version of the text (please note that Figure 4 has been modified to include new data).

-It is somehow redundant to put the Hh network twice in Fig. 1F and Fig. 3A.

We agree that it is somehow redundant. We decided to include a simple scheme (no parameters, no degradations...) as a support for the introduction of the pathway. It felt strange to refer to the first paragraphs of the introduction to a scheme in Figure 3 (which is there because it is where we first introduce the model). It also felt out of place to move the full scheme to figure one, because we felt that it was not as clear with all parameter names and production and degradation arrows. Again, the full scheme is required to illustrate what is the meaning of each parameter of the model.

During the design of the figures and the writing of the manuscript, we felt that both schemes (a simple one and a full one) provided valuable information (the first one as a support of the introduction, the second one as a support of the model).

In conclusion, we agree with the referee that both schemes show some information that is redundant, but since we have the extra space in Fig 1 underneath of Fig 1E, we think that keeping it helps us to write the introduction and to follow the pathway for readers not fully familiar with the Hh architecture.

Of course, if the referee disagrees and considers that the scheme in Fig 1 is not required, we can update figure 1 and remove it.

-Use nomenclature consistently, in Fig. 3A replace Cia for CiA.

We apologize for this typo. We submitted by mistake an earlier version of fig. 3 and 4 (this latter now fig. 5). In the correct final versions (uploaded as part of this new version), the nomenclature is consistent between text, figures and equations.

© 2022. Published by The Company of Biologists under the terms of the Creative Commons Attribution License (https://creativecommons.org/licenses/by/4.0/).

Second decision letter

MS ID#: DEVELOP/2021/199842

MS TITLE: Patterning on the move: the effects of Hh morphogen source movement on signaling dynamics

AUTHORS: David G Miguez, Antonella Iannini, Diana Garcia-Morales, and Fernando Casares

I have now received all the referees reports on the above manuscript, and have reached a decision. The referees' comments are appended below, or you can access them online: please go to BenchPress and click on the 'Manuscripts with Decisions' queue in the Author Area.

The overall evaluation is positive and we would like to publish a revised manuscript in Development, provided that the referees' comments can be satisfactorily addressed. All three referees acknowledge the revisions and clarifications you have provided and recognise the insight provided by your study. Referees 2 and 3 request further clarifications on several points and I would particularly draw your attention to the issue raised by Referee 3 about how Hh diffusion is described in the simulations.Please attend to all of the reviewers' comments in your revised manuscript and detail them in your point-by-point response. If you do not agree with any of their criticisms or suggestions explain clearly why this is so. If it would be helpful, you are welcome to contact us to discuss your revision in greater detail. Please send us a point-by-point response indicating your plans for addressing the referee’s comments, and we will look over this and provide further guidance.

Reviewer 1

Advance summary and potential significance to field

The manuscript elaborates on the important point that the temporal aspect of signalling is crucial in development.
In particular, it provides an interesting insight into the flexibility of Hh signalling pathway in performing different functions depending on the signal dynamics: patterning a tissue with a static source, or induce a cellular fate across entire tissues with a moving source.

Comments for the author

From what I could see in the reviewed manuscript and the responses to the reviewes, the questions and comments made in the first revision were carefully addressed by the authors.
I think that the manuscript is well written and presents interesting results. In the current version, the clarity of the presentation has been made clearer by the authors.
I would therefore recommend the publication of this work in Development.

Reviewer 2

Advance summary and potential significance to field

In this article Miguez et al explore how Hh signalling can mediate patterning information in different contexts, using a combination of experimental and computational approaches.

Comments for the author

It has been a little while since the previous review round and so I have read the MS with fresh eyes and I like the new version much better. The authors have made an effort to address most of my concerns. The article reads much better and the results are more convincing, particularly with the addition of new experiments and expansion of the parameters explored computationally. I have just a few remaining concerns that should be straight forward to address that I list below.

© 2022. Published by The Company of Biologists under the terms of the Creative Commons Attribution License (https://creativecommons.org/licenses/by/4.0/).

1. When the equations are introduced the authors state: “We model Smo as in a dynamic equilibrium in which production and degradation are equal and thus cancel each other, so there is no explicit degradation coefficient for Smo.”

I found this confusing. What do the authors mean by production and degradation being equal at dynamic equilibrium? For a species of concentration C that is produced at a constant rate and degraded linearly at rates \alpha and \beta respectively we have dC/dt = \alpha - \beta C so we would expect that at equilibrium C = \alpha / \beta. I do not follow the argument of equal degradation and production rates. Whether and when C reaches steady state depends on the value of \beta. Perhaps what the authors assume is that \beta is much larger for Smo relative to all other reactions in the system and so the baseline concentration of Smo is assumed to be constant? My understanding is that the authors indeed assume that Smo levels are non zero and fixed across the tissue and they only follow transitions between the bound and free state of Smo with Ptc. Is this right? How important is this assumption and how well founded? Why did the authors make this choice and what are the precise initial conditions for the spatial concentration of Smo? Does explicitly modelling production and degradation of Smo change the analysis? Is the requirement that Smo is at steady state essential for the conclusions of the model. I would suggest clarifying these issues both where the model is introduced and in more details in the methods section.

2. On page 5 the authors present an argument that explains the displaced peaks in CiA and Ptc levels in cases without a moving source. As far as I understand the explanation they provide is based on delays due to feedback between CiA and Ptc. One theoretical prediction wpuld be that the distance between the peaks should then be a function of \alpha_3 and k_3. It would be nice to see a rigorous analysis of the model that backs this prediction. This could be done by varying \alpha_3 and k_3 while keeping all other parameters fixed and computing the distance between the peaks which can be plotted as a heat map. I think this would make the verbal argument the authors present more quantitative and convincing.

3. I also wonder if the authors can predict what is the relevant timescale between the signalling dynamics and the movement of the furrow that would lead to collapsing peaks. For example on page 10 the authors state that signalling dynamics take about 60mins to reach steady state, what is the range of the speed of the MF that would give collapsing profiles for Ptc and CiA? The model should be able to predict how fast or slow the MF should move relative to the signalling dynamics and this can be used as a consistency check since the relative dynamics of the MF and Hh signalling are known.

4. A minor comment: in the parameter table concentration is sometimes denoted as concentration and others as conc, please use consistent notation. Also what is the difference between estimated and estimated from exp data?

Reviewer 3

Advance summary and potential significance to field

In their revised manuscript, the authors made an important effort to address the concerns of the prior round of review. While the hypothesis of the work is interesting and some of the concerns of the prior review were adequately addressed, in my opinion some issues remain to be resolved in order for the manuscript to be accepted for publication.

Comments for the author

1. An important aspect of the manuscript is the dynamics of the Hh gradient. In contrast to their first submission, in the revised version the authors use two equations for [Hh]. One is the spatial-dependent diffusion equation, while the other is part of a set of ODEs that are integrated in each cell. This presentation of the equations is inadequate and unacceptable. In their Materials and Methods section the authors state: “For each time step, the first equation for Hh diffusion is integrated globally in space and time. Due to the spatial gradient, the amount of Hh that each cell is receiving, Hh, depends on its position. This value Hh is computed for each cell and time step, as the average concentration for all pixels inside each cell. Next, the value of Hh is used to integrate

© 2022. Published by The Company of Biologists under the terms of the Creative Commons Attribution License (https://creativecommons.org/licenses/by/4.0/).

the six ODEs (see equations in the main text) independently for each of the 100 cells in the system.” From this presentation, it seems like there is a diffusible Hh morphogen that is not affected by degradation and Ptc binding, while there is another local Hh concentration that is indeed affected by these processes. Since the authors are solving the equations numerically anyways, I don’t see why Hh diffusion, degradation, and receptor binding are not included in a single equation (as in their original submission).

2. The main claim of the paper is that the coincidence in Ptc and CiA expression in the CE correspond to an ‘earlier point’ in the dynamics of the system. Their model reproduces this observation and the new addition to Figure 4 (c96>HhGFP overexpression experiment) also reproduces this observation, but does not show that the CE is interpreting the signal at an earlier time point. The authors also observe that Ptc levels are “significantly lower in the CE that in the other tissues,” so it is possible that Ptc and CiA coincide in the CE as a result of lower Ptc activation (that could be explained by a tissue-specific repressor of ptc in the CE). Could the authors run their simulations and show whether the CiA and Ptc peaks coincide at steady-state when ptc production (/alpha3) is significantly lower?

3. Figures are not adequately referenced in the text and this makes the manuscript very difficult to read. The authors should reference to each individual panel of the figure immediately after they present their results. For example, in page 3, add references where indicated: “However, we noted two things. First, the signaling profiles of Ptc and CiA in the wing, antenna and ocellus were qualitatively similar, with a first peak of Ptc followed by a peak in CiA further from the Hh source (ADD REFERENCE FIGURE PANEL HERE). On the other hand, the signaling signature of Hh in the CE was qualitatively different from the others, with a peak expression of Ptc that is coincident in space with a maximum in CiA (ADD REFERENCE FIGURE PANEL HERE). A second main difference is that the signal intensity of Ptc at this maximum was significantly lower in the CE that in the other tissues (ADD REFERENCE FIGURE PANELS THAT NEED TO BE COMPARED HERE).” The authors should not leave the reader the job of finding which figure and panel they are referring to.

Other comments:

- Reorganisation of figures. I suggest that the c96>HhGFP overexpression experiment should be separated from the atoRNAi experiment in Figure 4 and show more details (for example, in Fig. 4A, I cannot see extopic Ptc expression in the image even though they claim in Fig. 4B that there is; the authors may split the channels to show these details). If the authors what the paper to be published as a Research Report with 4 figures, I believe that Figure 1 is unnecessary or should be combined with Figure 2.
- Figure 4 is not adequately labeled. Panels should indicate what is the genotype. In the current presentation of this figure, two different experiments in two different organs are shown, but there are no labels for what is their genotype. Simply using ‘eye mutant’ is inadequate.

Minor typos:

- page 5, third paragraph, ‘is displaces’, should say ‘is displaced’
- page 5, third paragraph, ‘in a thin domain that crosses the wing pouch (Fig. 3)’, should say ‘along the DV boundary of the wing pouch (Fig. 4A)’
- page 9, bottom, ‘disk’, should say ‘disc’

Second revision

Author response to reviewers' comments

Reviewer 1. Advance Summary and Potential Significance to Field:
The manuscript elaborates on the important point that the temporal aspect of signalling is crucial in development. In particular, it provides an interesting insight into the flexibility of Hh signalling pathway in performing different functions depending on the signal dynamics: patterning a tissue with a static source, or induce a cellular fate across entire tissues with a moving source.

© 2022. Published by The Company of Biologists under the terms of the Creative Commons Attribution License (https://creativecommons.org/licenses/by/4.0/).

Reviewer 1 Comments for the Author:
From what I could see in the reviewed manuscript and the responses to the reviewes, the questions and comments made in the first revision were carefully addressed by the authors. I think that the manuscript is well written and presents interesting results. In the current version, the clarity of the presentation has been made clearer by the authors. I would therefore recommend the publication of this work in Development.

R1. Reviewer 1 is satisfied with the revised version. No specific comments need to be addressed.

Reviewer 2. Advance Summary and Potential Significance to Field:
In this article Miguez et al explore how Hh signalling can mediate patterning information in different contexts, using a combination of experimental and computational approaches.

Reviewer 2 Comments for the Author:
It has been a little while since the previous review round and so I have read the MS with fresh eyes and I like the new version much better. The authors have made an effort to address most of my concerns. The article reads much better and the results are more convincing, particularly with the addition of new experiments and expansion of the parameters explored computationally. I have just a few remaining concerns that should be straightforward to address that I list below.

1. When the equations are introduced the authors state: “We model Smo as in a dynamic equilibrium in which production and degradation are equal and thus cancel each other, so there is no explicit degradation coefficient for Smo.”

I found this confusing. What do the authors mean by production and degradation being equal at dynamic equilibrium? For a species of concentration C that is produced at a constant rate and degraded linearly at rates \alpha and \beta respectively we have dC/dt = \alpha - \beta C so we would expect that at equilibrium C = \alpha / \beta. I do not follow the argument of equal degradation and production rates. Whether and when C reaches steady state depends on the value of \beta. Perhaps what the authors assume is that \beta is much larger for Smo relative to all other reactions in the system and so the baseline concentration of Smo is assumed to be constant? My understanding is that the authors indeed assume that Smo levels are non zero and fixed across the tissue and they only follow transitions between the bound and free state of Smo with Ptc. Is this right? How important is this assumption and how well founded? Why did the authors make this choice and what are the precise initial conditions for the spatial concentration of Smo? Does explicitly modelling production and degradation of Smo change the analysis? Is the requirement that Smo is at steady state essential for the conclusions of the model. I would suggest clarifying these issues both where the model is introduced and in more details in the methods section.
R1. We apologize for the poor explanation of this assumption of the model. Indeed, we assume a non-zero, constant Smo concentration across the tissue which only undergoes transitions between the unbound and Ptc-bound forms, as the referee suggests, with Smo production (\alpha) being equal to its degradation (\beta \times C). As for whether a constant Smo is a good biological assumption: Since the mechanisms of Smo regulation by Ptc are still not clear, we decided to adopt this simplification since otherwise, the mounting in parameter numbers would make the interpretation of the model more cumbersome. In this form, we think it captures the core effect of the feedback regulation. Earlier versions of the model included explicit production (\alpha) and degradation (\beta Smo) for Smo. In these versions of the model, using values for production and degradation in the range of the rest of the molecules, after a fast transient (few minutes), the levels of total Smo (free+bound) remained constant (since Smo is not affected by the rest of molecules of the feedback). Therefore, based on these previous versions of the model, we decided to simplify the equation of Smo, since this transient was not significantly affecting the dynamics, and we felt that a model focused on the interactions in the feedback loop will be much clearer and simpler to understand.
We have included in the present version of the manuscript an explanation of why we dynamics of production and degradation of Smo is not explicitly modeled, following this suggestion from the referee.

© 2022. Published by The Company of Biologists under the terms of the Creative Commons Attribution License (https://creativecommons.org/licenses/by/4.0/).

2. On page 5 the authors present an argument that explains the displaced peaks in CiA and Ptc levels in cases without a moving source. As far as I understand the explanation they provide is based on delays due to feedback between CiA and Ptc. One theoretical prediction would be that the distance between the peaks should then be a function of \alpha_3 and k_3. It would be nice to see a rigorous analysis of the model that backs this prediction. This could be done by varying \alpha_3 and k_3 while keeping all other parameters fixed and computing the distance between the peaks which can be plotted as a heat map. I think this would make the verbal argument the authors present more quantitative and convincing.
R2. Following the suggestion of the referee, we performed numerical simulations where we measured the distance between peaks changing the two parameters inside the feedback loop. We constructed a 3D heat map, shown below.

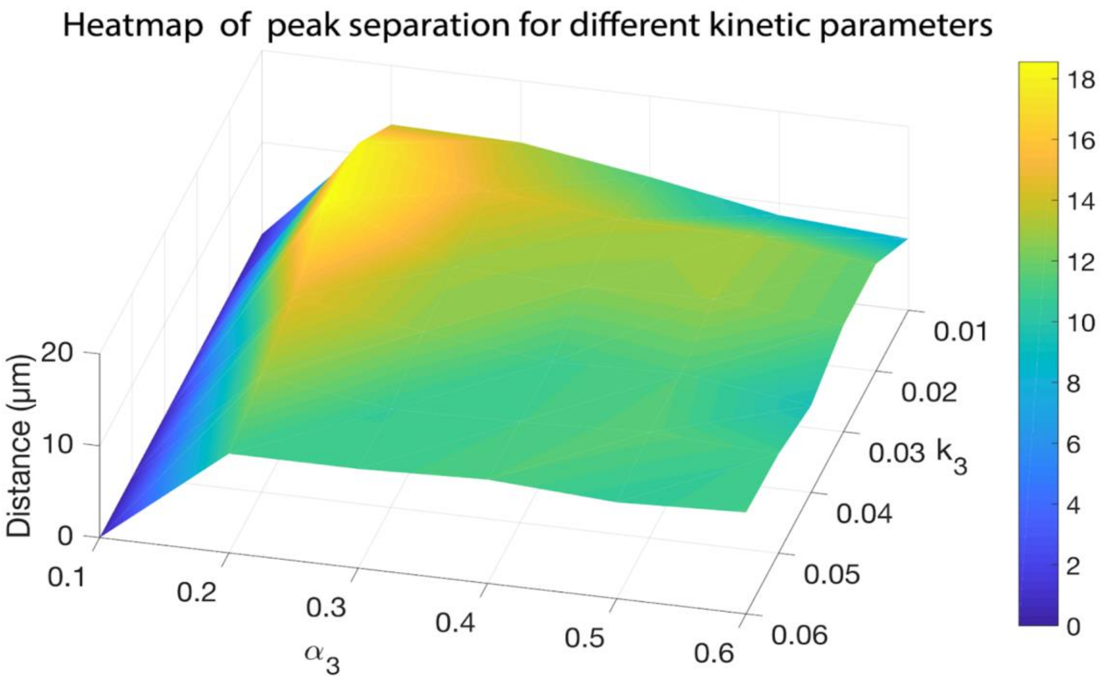

The vertical axis and the color bar represent the distance between CiA and Ptc peaks. Our results show that a slower feedback (low alpha3 and low k3) results in increased distance between peaks. This occurs because, if the feedback takes longer to operate, the Hh gradient is able to reach further in the system, inducing activation of more distant cells and therefore displacing the peaks far from the source.

A faster dynamics only produces that the steady state is reached faster (in fact, distance for alpha3 = 0.1 is zero because there was no time for the peaks to separate in 300 minutes). This is related to simulations suggested by referee 3 (see below), where we show that for low alpha_3 reaching the steady state takes longer (around 600 min), and peaks become very separated (panel 4 in figure below).

New Suppl. Fig. 5B: Effects of reducing \alpha_3 (from left to right) on time to reaching steady state and peak separation (result of a single simulation, not averaged over 10 simulations).

© 2022. Published by The Company of Biologists under the terms of the Creative Commons Attribution License (https://creativecommons.org/licenses/by/4.0/).

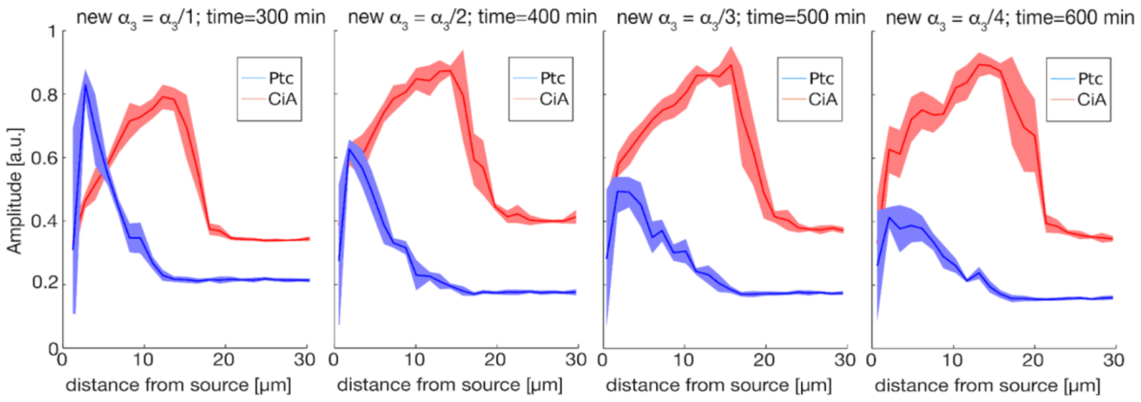

We sincerely thank the referee for the suggestion of performing this analysis, that showed us that the temporal dynamics of the signaling pathway and the separation of the peaks of the critical molecules, Ptc and CiA, depend on the relation between two temporal scales: those of Hh diffusion and the negative feedback.

This figure now is part of the manuscript as Supp. Figure 5A, and an explanation of these findings has been added to the discussion.

3. I also wonder if the authors can predict what is the relevant timescale between the signalling dynamics and the movement of the furrow that would lead to collapsing peaks. For example on page 10 the authors state that signaling dynamics take about 60 mins to reach steady state, what is the range of the speed of the MF that would give collapsing profiles for Ptc and CiA? The model should be able to predict how fast or slow the MF should move relative to the signaling dynamics and this can be used as a consistency check since the relative dynamics of the MF and Hh signaling are known.
R3. The sentence in page 10 refers to the dynamics of Hh only (diffusion + constant degradation); the rest of the signaling, for the parameter values here used, takes around 300 min. We have used the information available on the dynamics of MF movement and Hh signaling to check whether the model predicts correctly their relative dynamics. The MF moves about 1 cell every 60-100 minutes (at 25ºC; Basler and Haffen, 1989). The Hh signaling pathway completes its feedback (i.e. a cell will express high Ptc and low CiA) in 360 min at 18ºC (as in Nahmad and Stathopoulos, 2009, a value obtained for the wing disc), which translates to about 180 min at 25ºC (using estimates of developmental rates by Powsner, 1935). Therefore, a cell just anterior to the MF will experience the highest Hh levels for just ⅓ of the time required to reach full signaling -too short for full signaling. In our simulations (see Figure 2 C,D), full signaling happens at 300 min and indeed, at t = 60 min (<100 min), the CiA and Ptc peaks coincide, while at t = 200 min (> 100 min) the peaks already show the beginning of their separation. We note that, serendipitously, the MF speed in *ey>ato-RNAi* is reduced to a third of normal, a situation in which we detect an incipient, but significant, peak separation. So we believe that our model (with the parameter set we have used) captures well also the relative time scales of MF and Hh signaling dynamics. We have included a note on the relative dynamics of MF movement and Hh signaling in the text, preceding the experiment of MF retardation, to describe these estimates.

4. A minor comment: in the parameter table concentration is sometimes denoted as concentration and others as conc, please use consistent notation.
R4.1 Done.

Also what is the difference between estimated and estimated from exp data?
R4.2. We apologize for the confusion. We refer to “estimated from exp data” when we use experimental data to indirectly obtain the value of a given parameter, and we used “estimated” for parameters that we adjust in the model to fit a certain behavior that we know is happening experimentally. For instance, we calibrate the Hill coefficients because we know the transition between active and inactive should occur around concentration values of the protein involved. To avoid confusion, we now refer in the text to these parameters as “calibrated”.

© 2022. Published by The Company of Biologists under the terms of the Creative Commons Attribution License (https://creativecommons.org/licenses/by/4.0/).

Reviewer 3 Advance Summary and Potential Significance to Field:
In their revised manuscript, the authors made an important effort to address the concerns of the prior round of review. While the hypothesis of the work is interesting and some of the concerns of the prior review were adequately addressed, in my opinion some issues remain to be resolved in order for the manuscript to be accepted for publication.

Reviewer 3 Comments for the Author:
1. An important aspect of the manuscript is the dynamics of the Hh gradient. In contrast to their first submission, in the revised version the authors use two equations for [Hh]. One is the spatial-dependent diffusion equation, while the other is part of a set of ODEs that are integrated in each cell. This presentation of the equations is inadequate and unacceptable. In their Materials and Methods section the authors state: “For each time step, the first equation for Hh diffusion is integrated globally in space and time. Due to the spatial gradient, the amount of Hh that each cell is receiving, Hh, depends on its position. This value Hh is computed for each cell and time step, as the average concentration for all pixels inside each cell. Next, the value of Hh is used to integrate the six ODEs (see equations in the main text) independently for each of the 100 cells in the system.” From this presentation, it seems like there is a diffusible Hh morphogen that is not affected by degradation and Ptc binding, while there is another local Hh concentration that is indeed affected by these processes. Since the authors are solving the equations numerically anyways, I don’t see why Hh diffusion, degradation, and receptor binding are not included in a single equation (as in their original submission).

R.1. We sincerely apologize for the poor explanation of how the Hh concentration is being computed.
To simplify the set of equations and make them more readable (following a suggestion from a referee in the previous round of revision) we eliminated the subindexes and separated processes computed globally (Hh production at the source and Hh diffusion) and processes computed locally (activation and repression in the signaling cascade downstream of Hh, binding of Hh to Ptc, degradation of the complex…). Since Hh is involved in both local and global processes, this resulted in two equations for Hh (one PDE and one ODE), which is inadequate, as the referee indicates. In the present version of the text we have updated the set of equations to compile the local and global processes of Hh in a single equation (see below).

$$
\begin{aligned}
\frac{\partial [Hh]}{\partial t} &= \theta(\vec{r},\alpha_1) + D\frac{\partial^2 [Hh]}{\partial \vec{r}^2} + k_2[HhPtc] - k_1[Hh][Ptc] - \beta_1[Hh] \\
\frac{\partial [HhPtc]}{\partial t} &= k_1[Hh][Ptc] - k_2[HhPtc] - \beta_3[HhPtc] \\
\frac{\partial [Ptc]}{\partial t} &= -k_1[Hh][Ptc] + k_2[HhPtc] - k_3[Smo][Ptc] + k_4[SmoPtc] - \beta_3[Ptc] + \frac{\alpha_3[CiA]^m}{[CiA]^m + M_3^m} \\
\frac{\partial [Smo]}{\partial t} &= -k_3[Smo][Ptc] + k_4[SmoPtc] \\
\frac{\partial [SmoPtc]}{\partial t} &= k_3[Smo][Ptc] - k_4[SmoPtc] \\
\frac{\partial [CiA]}{\partial t} &= \frac{\alpha_2[Smo]^m}{[Smo]^m + M_2^m} - \beta_2[CiA]
\end{aligned}
$$

The referee also raised an important issue, pointing out that from our explanation in the Methods, it seemed that Hh is not affected by degradation and Ptc binding. We apologize for this, and edited the explanation in this section to clarify that the levels of Hh that are computed globally are of course affected by the local interactions (binding to Ptc, degradation…).
Basically, these levels are computed for each iteration as follows.

- (1) Hh values are stored in a 2D matrix of 55x55 pixels (named HH_{global});

© 2022. Published by The Company of Biologists under the terms of the Creative Commons Attribution License (https://creativecommons.org/licenses/by/4.0/).

- (2) For each iteration, the level of Hh diffuses globally (a new HH_{global} is computed);
- (3) Next, the amount of Hh that each cell receives is computed (from HH_{global} we obtain Hh_i);
- (4) Next, each Hh_i changes locally due to interactions with the receptor (binding, unbinding, degradation);
- (5) Next, the new values of Hh_i are used to compute the values of a new matrix Hh_{global} (so, levels of the diffusing molecule are determined by the events in each cell).

We thank the referee for his/her suggestion, and we sincerely hope that the updated Methods and equations in the present version of the manuscript are both accurate and clear.

2. The main claim of the paper is that the coincidence in Ptc and CiA expression in the CE correspond to an ‘earlier point’ in the dynamics of the system. Their model reproduces this observation and the new addition to Figure 4 (c96>HhGFP overexpression experiment) also reproduces this observation, but does not show that the CE is interpreting the signal at an earlier time point. The authors also observe that Ptc levels are “significantly lower in the CE that in the other tissues,” so it is possible that Ptc and CiA coincide in the CE as a result of lower Ptc activation (that could be explained by a tissue-specific repressor of ptc in the CE). Could the authors run their simulations and show whether the CiA and Ptc peaks coincide at steady-state when ptc production (/alpha3) is significantly lower?

R.2. The referee raises an interesting question suggesting that maybe the differences in patterning come from the expression levels of Ptc between the different tissues. To test this, the referee proposes to take advantage of the model and run simulations with reduced levels of Ptc production (\alpha_3). Following her/his suggestion, we run numerical simulations with decreasing values of \alpha_3. Results are summarized in the figure below, with \alpha_3 reducing from left to right (result of a single simulation, not averaged over 10 simulations)

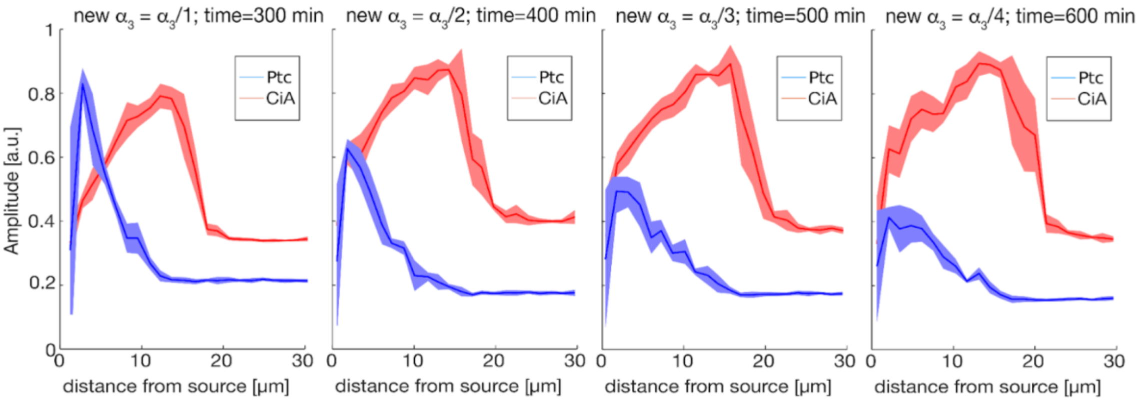

The model predicts that peak separation takes place in the system, but it occurs at later time points. This makes sense, since reducing the rate of production of alpha_3 also slows down the dynamics, so it is expected that everything occurs later in time. In addition, lower alpha_3 results in even more separation between peaks (related to the comment by referee 2, and the heatmap in the new Supp. fig 5A). Smaller values of alpha_3 (below ⅙ of its original value) result in almost constant levels of Smo across the system, so CiA levels become also constant. We thank the referee again for this suggestion, in the direction of a comment from referee 2 (test parameter values).

3. Figures are not adequately referenced in the text and this makes the manuscript very difficult to read. The authors should reference to each individual panel of the figure immediately after they present their results. For example, in page 3, add references where indicated: “However, we noted two things. First, the signaling profiles of Ptc and CiA in the wing, antenna and ocellus were qualitatively similar, with a first peak of Ptc followed by a peak in CiA further from the Hh source (ADD REFERENCE FIGURE PANEL HERE). On the other hand, the signaling signature of Hh in the CE was qualitatively different from the others, with

© 2022. Published by The Company of Biologists under the terms of the Creative Commons Attribution License (https://creativecommons.org/licenses/by/4.0/).

a peak expression of Ptc that is coincident in space with a maximum in CiA (ADD REFERENCE FIGURE PANEL HERE). A second main difference is that the signal intensity of Ptc at this maximum was significantly lower in the CE that in the other tissues (ADD REFERENCE FIGURE PANELS THAT NEED TO BE COMPARED HERE)." The authors should not leave the reader the job of finding which figure and panel they are referring to.

R.3. We apologize for this. We have now cited the Figures as indicated throughout the paper.

Other comments:

- Reorganisation of figures. I suggest that the c96>HhGFP overexpression experiment should be separated from the atoRNAi experiment in Figure 4 and show more details (for example, in Fig. 4A, I cannot see extopic Ptc expression in the image even though they claim in Fig. 4B that there is; the authors may split the channels to show these details). If the authors what the paper to be published as a Research Report with 4 figures, I believe that Figure 1 is unnecessary or should be combined with Figure 2.
We have followed the reviewer's suggestion and reorganized the figures, such that while preserving a total of four, the c96/wing experiment and the MF-retardation experiment and simulation are split into independent figures (3 and 4, respectively) and figure 1 and 2 are condensed in a single figure now.
- Figure 4 is not adequately labeled. Panels should indicate what is the genotype. In the current presentation of this figure, two different experiments in two different organs are shown, but there are no labels for what is their genotype. Simply using 'eye mutant' is inadequate.
Now in the new Figure 3, the genotype is indicated on one of the panels. In figure 4, the "mutant" genotype is really a mutant genotype *plus* a temperature treatment, so we have decided to leave the description to the figure legends and to panels D, where the control and mutant fly phenotype is shown.

Minor typos:

- page 5, third paragraph, 'is displaces', should say 'is displaced'. Amended.
- page 5, third paragraph, 'in a thin domain that crosses the wing pouch (Fig. 3)', should say 'along the DV boundary of the wing pouch (Fig. 4A)'. Amended.
- page 9, bottom, 'disk', should say 'disc' . Amended.

Third decision letter

MS ID#: DEVELOP/2021/199842

MS TITLE: Patterning on the move: the effects of Hh morphogen source movement on signaling dynamics

AUTHORS: David G Miguez, Antonella Iannini, Diana Garcia-Morales, and Fernando Casares
ARTICLE TYPE: Research Report

I am happy to tell you that your manuscript has been accepted for publication in Development, pending our standard ethics checks.

© 2022. Published by The Company of Biologists under the terms of the Creative Commons Attribution License (https://creativecommons.org/licenses/by/4.0/).
